# Discrepancies in occupancy and abundance approaches to identifying and protecting habitat for an at‐risk species

**DOI:** 10.1002/ece3.3131

**Published:** 2017-06-15

**Authors:** Reilly R. Dibner, Daniel F. Doak, Melanie Murphy

**Affiliations:** ^1^ Program in Ecology University of Wyoming Laramie WY USA; ^2^ Environmental Studies Program University of Colorado, Boulder Boulder CO USA; ^3^ Department of Ecosystem Science and Management Program in Ecology University of Wyoming Laramie WY USA

**Keywords:** abundance, conservation, environmental limits, horned lizard, occupancy, *Phrynosoma hernandesi*

## Abstract

Predicting how environmental factors affect the distribution of species is a fundamental goal of conservation biology. Conservation biologists rely on species distribution and abundance models to identify key habitat characteristics for species. Occupancy modeling is frequently promoted as a practical alternative to use of abundance in identifying habitat quality. While occupancy and abundance are potentially governed by different limiting factors operating at different scales, few studies have directly compared predictive models for these approaches in the same system. We evaluated how much occupancy and abundance are driven by the same environmental factors for a species of conservation concern, the greater short‐horned lizard (*Phrynosoma hernandesi*). Occupancy was most strongly dictated by precipitation, temperature, and density of ant mounds. While these factors were also in the best‐supported predictive models for lizard abundance, the magnitude of the effects varied, with the sign of the effect changing for temperature and precipitation. These discrepancies show that while occupancy modeling can be an efficient approach for conservation planning, predictors of occupancy probability should not automatically be equated with predictors of population abundance. Understanding the differences in factors that control occupancy versus abundance can help us to identify habitat requirements and mitigate the loss of threatened species.

## 1 INTRODUCTION

Habitat degradation and loss are increasing the rate of global biodiversity declines, a trend that is expected to accelerate throughout the next century (Butchart et al., 2010). Changes in land use that degrade environmental quality will increase the number of species potentially threatened by human activities, and land use change is predicted to have the biggest effect on terrestrial ecosystems by the year 2100 (Sala et al., 2000). In the face of these changes, developing reliable, simple, and biologically relevant models to predict where species will occur and how abundant they are across natural and modified landscapes are a pressing research need in conservation biology (e.g. Elith & Leathwick, 2009; Dawson, Jackson, House, Prentice, & Mace, 2011).

Site occupancy models offer one such approach to quantifying the distribution patterns of species; occupancy models predict the probability that a species is present at a site. Occupancy models are elegant in their use of simple presence–absence data and biologically relevant in their ability to incorporate imperfect detection, multiple species and seasons, and covariates for surveys and sites across multiple habitat types (MacKenzie, 2006). Occupancy models have garnered much attention due to their potential to provide a reliable and cost‐effective method of analyzing population distribution and habitat use, especially compared with methods that attempt to estimate population abundance (Noon, Bailey, Sisk, & Mckelvey, 2012; Tempel & Gutiérrez, 2013; Casner, Forister, Ram, & Shapiro, 2014). For rare, elusive, or sensitive species, the difficulty of gathering accurate abundance data makes occupancy models especially promising.

These advantages have led researchers to use occupancy model results—predictions of the probability of occupancy—to draw conclusions about presumed abundance patterns (e.g. Tempel & Gutiérrez, 2013; Casner et al., 2014). While this focus on occupancy is likely because the state variable is easier to monitor than abundance, the scale of observation can affect the results of occupancy analyzes (MacKenzie, 2006; Ellis, Ivan, & Schwartz, 2014; Wilson & Schmidt, 2015); one explanation for this sensitivity is that occupancy and abundance can be determined by environmental characteristics that operate at different temporal and spatial scales (Orrock, Pagels, McShea, & Harper, 2000) and may be dictated by different processes (Cingolani, Cabido, Gurvich, Renison, & Díaz, 2007). Only direct comparisons of statistical approaches to quantify occupancy and abundance can indicate how reliable occupancy analyzes are for characterizing abundance patterns, but we currently have surprisingly few such tests (Royle & Nichols, 2003; Sileshi, 2007; Couturier, Cheylan, Bertolero, Astruc, & Besnard, 2013), and the focus has been on temporal variation (but see Fletcher, MacKenzie, & Villouta, 2005; Wilson & Schmidt, 2015). Understanding this relationship for rare or habitat‐limited species could be especially valuable for conservation efforts across temporal and spatial scales.

**Figure 1:**
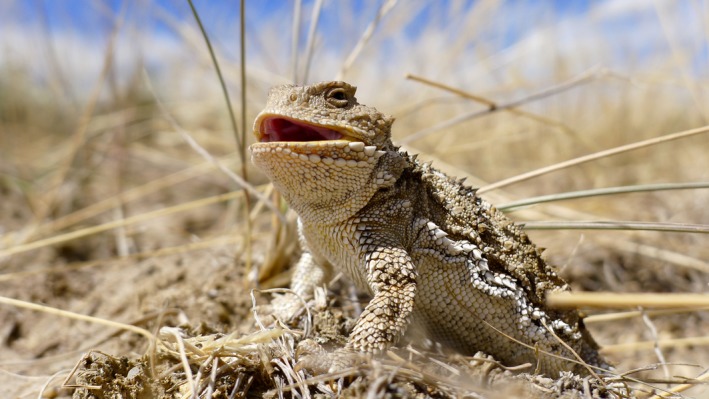
Greater short‐horned lizards (*Phrynosoma hernandesi*) are a sensitive species found throughout the sagebrush and shortgrass ecoregions of western North America and are a Species of Greatest Conservation Need in Wyoming (Wyoming Game and Fish Department 2010)

We performed such a comparison with respect to spatial scale using the greater short‐horned lizard (*Phrynosoma hernandesi*) as our focal species (Figure 1). This lizard is broadly dispersed throughout arid western North American ecosystems (Hammerson, 2007), but in many areas populations are exposed to mounting pressure as energy development (Copeland, Pocewicz, & Kiesecker, 2011; Souther et al., 2014) and other human use impacts expand into their sagebrush habitat. As a whole, the horned lizards (genus *Phrynosoma*), appear to be highly sensitive to human‐caused habitat degradation (e.g. Beauchamp, Wone, Bros, & Kutilek, 1998). Understanding what environmental factors determine greater short‐horned lizard occurrence and abundance will improve our ability to predict what factors are most critical to preserve populations of this species and related species.

We studied the greater short‐horned lizard in relatively undisturbed areas to address two general questions concerning the use of occupancy modeling to identify good predictors of local habitat quality:

1. Which habitat factors best predict horned lizard occupancy and which best predict abundance?
2. How similar are these environmental factors and their relationships with hornd lizard occupancy and abundance?

We discuss our results in light of their implications for conservation management and emphasize how differences between occupancy and abundance results could undermine effective conservation monitoring and planning, if not recognized. While we discuss the specific implications for greater horned lizards, we use the results to draw broader inferences about the use of occupancy and abundance data to predict species vulnerability to anthropogenic disturbances, and how to craft successful conservation management strategies.

## 2 METHODS

To answer our questions, we did three things: (1) Surveyed for lizard occurrence and abundance in widely distributed plots, as well as quantified multiple climate and habitat characteristics of these sites; (2) Conducted separate analyzes to predict occurrence and abundance based on these environmental variables; and (3) Compared the effects of environmental factors revealed in these occupancy vs. abundance models.

### 2.1 Study area

Greater short‐horned lizards inhabit high desert and arid montane environments in western North America from northern Mexico to southern Canada. Our study sites spanned the large portion of the greater short‐horned lizard's range that is dominated by sagebrush (*Artemesia* spp.), an ecosystem that has been fragmented, reduced in area (Walston, Cantwell, & Krummel, 2009), and has recently come under accelerated threat due to rapid energy development (e.g. Naugle et al., 2011). To select plots, we used a random stratification across temperature and precipitation gradients to characterize potential study regions that spanned the entire state of Wyoming, USA, but that had similar vegetation composition (Comer et al., 2003). These regions were undeveloped portions of Wyoming of about 1,000 km^2^ in area. We then selected three to four random points within each region and identified three to four plot locations per randomly selected point (50 × 100 m) in apparently undisturbed sagebrush habitats (≥1 km) from anthropogenic features (NatureServe 2014) within 1 km of the selected point for a total of 10–12 plots/region. We constrained plot locations to maintain a minimum distance to anthropogenic features (≥1 km) (NatureServe 2014), minimum distance between plots (≥1 km), similar slope gradients (<5%), and accessibility (≤1.6 km from shelter for lightning safety). This selection resulted in one hundred 50 × 100 m plots (Fig. S1, Table S1). Eighty‐nine of these plots were in relatively undisturbed environments and were the focus of this study.

### 2.2 Data collection

We surveyed 50 plots in 2012 (20 of which we resurveyed that season), 50 in 2013 (8 of which we resurveyed that season), and 12 plots in both years. In each plot we quantified: lizard presence or absence (occupancy); lizard density per hectare; prey availability; and vegetation cover. We quantified horned lizard occupancy of each plot by visually searching for lizards and fresh fecal pellets, which are distinct and easily identifiable for this species. Raw data on abundance were based on visual searches for lizards (fecal pellets are not a reliable indicator of lizard abundance; Beauchamp et al., 1998). For each survey, two to three people walked approximately two meters apart, with the person on the interior edge of the plot placing survey flags to guide survey lines on subsequent passes. Surveys were complete when we covered all ground along both cardinal axes, east to west and north to south, such that all area within a plot was covered twice. A complete survey took between 75 and 240 min, depending on the number of lizards encountered.

For each lizard encountered we recorded a location via GPS (Garmin^®^ GPSMAP^®^ 76CSx, accuracy ± 5 m). Captured lizards were marked ventrally with unique numbers using a Sharpie^®^ marker. This temporary mark allowed us to identify lizards that we had already encountered during that survey. We recorded time, air temperature, and average wind speed (Kestrel^®^ 2000 Wind Meter) at the beginning and the end of surveys. We resurveyed 28 (20 in 2012, 8 in 2013) of the 89 plots 4–6 weeks after the first survey to quantify detection probability (MacKenzie, 2006).

We quantified information on invertebrate prey by leaving three 150 ml un‐baited pitfall traps at each plot for 24 hr (Griffin, 2003) and mapping all large ant mounds. Thatcher ants (*Formica obscuripes*) and western harvester ants (*Pogonomyrmex occidentalis*) form large mounds in the region; other horned lizard species feed preferentially on congeners of the western harvester ant (Rissing, 1981). The 24‐hr pitfall trap sampling period was not ideal for capturing rare ants (Borgelt & New, 2006), but we were primarily interested in characterizing the dominant available prey that lizards would most likely encounter. We used the Shannon diversity index to quantify diversity of available prey in pitfall traps (Shannon, 1948).

To quantify vegetation cover in each plot, we photographed 0.25 m^2^ quadrats at 10‐m intervals along two 50 m transects for a total of 10 photographs per plot (Parravicini et al., 2009). Photographs were taken perpendicular to the ground, and the low sagebrush (*Artemesia tridentata*) plants within these plots were generally ≥0.25 m^2^ in area. For each image, we categorized vegetation (sage, grass, soil, shrub, forb, other) at each of 16 regularly spaced points and quantified vegetation cover for each plot by calculating the proportion of intersections for each cover type.

We measured temperature at each plot for 1 year, in either 2012–2013 or 2013–2014 with iButton^®^ Thermochron^®^ temperature loggers buried in soil in plastic containers filled with silica (Dallas Semiconductor Corporation, Dallas, TX, USA), taking readings every 255 min to calculate annual and monthly temperature metrics (mean annual mean, standard deviation, minimum, and maximum, temperature). We used climate data (mean annual precipitation) for each plot downloaded from the WorldClim dataset (Hijmans, Cameron, Parra, Jones, & Jarvis, 2005; http://www.worldclim.org/bioclim, accessed October 3, 2014). In the final analyzes, we used iButton data for temperature and WorldClim data for precipitation.

All surveys were completed with approval from the University of Wyoming Animal Care and Use Committee (IACUC protocol 20140515RD00100‐01) and the Wyoming Game and Fish Department under a Chapter 33 scientific permit (33‐825).

### 2.3 Occupancy modeling

To identify the best predictors of horned lizard occupancy of plots, we created a set of single season, single species hierarchical occupancy models (MacKenzie, Nichols, Hines, Knutson, & Franklin, 2003) in R using the package RPresence (MacKenzie & Hines, 2014). We pooled data from 2012 to 2013 to increase analytical power, and used our multiple season surveys primarily to support this approach; if observations in 2013 were not strongly correlated with 2012 observations, we would not have pooled data in this way (see 3). The occupancy models included two parts: (1) habitat covariates for predicting probability of occupancy that may differ among sites but remain constant between surveys (occupancy ψ; MacKenzie et al., 2002) and (2) survey covariates that may vary over time, affecting the probability of observing lizards (detection *p*). To structure the occupancy models, we first identified the best covariates on detection; for this step, we held the probability of plot occupancy (ψ) constant and varied combinations of all survey‐specific variables (Table S2). Detection can vary substantially among species and accounting for variation in detection is important in modeling occupancy (Royle & Nichols, 2003; MacKenzie, 2006), particularly if detection is variable among sites. We selected the detection covariates that produced the model with the lowest AICc score for constant occupancy and used these covariates for the detection component in testing models to predict occupancy.

To build the set of candidate models for occupancy, we included all subset combinations of models that contained the following variables: an interaction between temperature and precipitation, ant mound density, ant diversity, diversity of nonant invertebrates, diversity of all invertebrates, percentage of ground area covered with soil, and percentage of ground area covered with sage (see Table S3 for full model list). We suspected that temperature and precipitation could interact in ways that they might affect aspects of lizard habitat that we could not measure directly; we included models with and without temperature and precipitation interactions. We also included ant abundance and ant diversity both with and without an interaction, as a simultaneous study of horned lizard diet indicated that diet diversity could vary across the geographic range of our study. The best‐supported predictive model had the lowest AICc score. We measured the importance of each variable by its summed AIC weight (AICω) across the model set.

To assess goodness of fit of the models to the data, we calculated a Pearson chi‐square test to observed and bootstrapped data (MacKenzie & Bailey, 2004). To test overall model sensitivity to detection probabilities, we ran the model set with different covariates on detection (Royle, 2006).

### 2.4 Abundance modeling

To generate abundance estimates, we initially used Lincoln mark–recapture data (Lincoln, 1930) collected from our 28 resurveyed plots to derive plot‐specific population abundance estimates. Extrapolating Lincoln mark–recapture data to additional survey plots can be problematic, potentially resulting in inaccurate abundance estimates (Pocock, Frantz, Cowan, White, & Searle, 2004), but population estimates generated from within‐survey data can be useful if they correlate strongly with more formal mark–recapture estimates. We found that bias‐corrected Chao estimates for population size (time variation model [equation 10] in Chao, 1989; Keating, Schwartz, Haroldson, & Moody, 2002) were extremely well correlated with Lincoln mark–recapture estimates (Spearman ρ = .88), so we used the Chao estimates as our dependent variable. While these estimates carry inherent uncertainty, we were most concerned with how environmental factors predicted observed *relative* abundances (hereafter “abundance”). High recapture and detection rates gave us confidence in our observations. We tested covariates for normality and transformed data to normalize the distribution, where relevant.

We first ran linear regression models to predict lizard abundance, running these models only on plots where lizards were found in our surveys. This approach to evaluating abundance data conditionally, without including zeros, has precedence (e.g. Fletcher et al., 2005) and is particularly relevant for conservation because occupancy and abundance may be separately determined by different environmental factors; understanding those differences can affect management and/or policy decisions. We used the same model set that we generated for occupancy models, including the survey variables as nuisance variables, plus the top models again without the nuisance variables (Table S4). We identified the best‐supported model and measured variable importance using AIC approaches, and identified the sign of relationships with abundance in the same way described above for occupancy models. To assess goodness of fit of the models, we calculated the coefficient of determination (*R* ^2^) for each model.

We further tested how occupancy versus abundance could give different results by applying an abundance analysis that included zeros. We ran linear mixed effects Poisson regression models that included the fixed effects that we used in the linear abundance regressions, with survey plot as a random effect. The use of this random effect accounts implicitly for observation error and accommodates overdispersion (i.e. zero‐inflation) within a maximum‐likelihood framework (Elston, Moss, Boulinier, Arrowsmith, & Lambin, 2001; Harrison, 2014). We evaluated model performance as described above.

### 2.5 Comparing approaches

We assessed potential mismatch between predictors of occupancy and abundance in several ways. First, we used the best‐supported model for the occupancy and linear regression abundance approaches to generate predictions across the observed range of each environmental variable, while holding all other independent variables constant at either their mean values, or at representative low or high values (first and third quartiles of empirical range, respectively) to account for interaction effects. To assess the robustness of these results, we also ran these predictions for the top five models of occupancy and for the parallel models for lizard abundance. We used the covariance matrix for fitted coefficients in the best model for abundance to select 1,000 random sets of coefficients, and used these to generate 95% confidence intervals for abundance predictions; we made parallel confidence intervals on the occupancy predictions using a bootstrap approach. For the bootstrap, we sampled randomly from our data to create new synthetic data and ran occupancy analysis on the top model using the synthetic data. We stored the model coefficients and then used these coefficients to predict occupancy estimates using our empirical data. We repeated this process 1,000 times for all variables of interest.

We also compared the sign and AIC weights (AICω) for environmental variables supported in models for occupancy vs. abundance (both with and without zeros included), and quantified the strength of the correlation between the predicted occupancy probability or abundance and each environmental predictor, as described above.

Finally, we plotted the predicted occupancy probability versus (1) the predicted lizard abundance for each study plot; (2) the product of these values (the predicted mean abundance corrected for probability of occupancy, which gives the predicted mean abundance in a plot, accounting for whether the site is likely to be occupied or not); and (3) the predicted abundance based on the mixed effects model analysis with plot as the random affect. Correspondence between occupancy probability and each of these three measures should have been high if occupancy predicted abundance well.

## 3 RESULTS

Lizards were observed in 60 of the 89 plots, with an average of 2.9 (±3.2 *SD*) individuals captured where found. Univariate relationships between environmental factors and the number of lizards in all plots, including those with no lizards, showed only weak patterns (Fig. S2). These poor relationships were largely due to the many interactions among covariates in their effects on occupancy and abundance, as we discuss below.

Naive detection of lizards was the same (either 0 or 1) in 25 of the 28 resurveys, and lizard and scat abundances were consistent among surveys (Spearman's Rank Correlation ρ = .622 and ρ = .683, respectively). Detection was high (0.89 [±0.06 *SD*]) across all plots (see Table S2 for full detection results), and the mean probability of site occupancy was 0.67 (±0.08 *SD*).

The best model for occupancy probability included an interaction between annual mean precipitation and annual mean temperature, plus an interaction between ant mound density and diversity of available ants (Table 1, Table S3). Survey temperature influenced detection (Table 1, Table S2). The top model showed a reasonable fit to the data (Figure S3).

**Table 1:** Best‐supported models for occupancy and lizard abundance, top four models for each approach are shown

Approach	Model structure^a^	ΔAIC_c_ b	AICω^b^	NLL	Model Fit^c^
Occupancy	amt*amp + ants*ant.h	0	1.76E‐01	−96.47	χ^2^ = 6.5, *p* > 0.5
Occupancy	amt*amp + ants*ant.h + other.h	1.83	9.21E‐02	−99.58	χ^2^ = 5.16, *p* > 0.5
Occupancy	amt*amp + ants	2.00	4.12E‐02	−95.67	χ^2^ = 5.61, *p* > 0.5
Occupancy	amt*amp + ants*ant.h + sage	2.46	6.72E‐02	−95.76	χ^2^ = 4.98, *p* > 0.5
Abundance	amt + amp + ants	0	1.26E‐01	−68.82	*R* ^2^ = 0.230
Abundance	amt*amp + ants	1.88	9.28E‐02	−67.73	*R* ^2^ = 0.230
Abundance	amt + amp + ants + sage	2.12	4.06E‐02	−68.56	*R* ^2^ = 0.216
Abundance	amt*amp + ants + soil	2.13	3.73E‐02	−68.65	*R* ^2^ = 0.216
GLMM	ants + all.h|survey plot	0	8.01E‐02	−192.24	Pseudo *R* ^2^ = 0.244
GLMM	ants	0.74	5.55E‐02	−193.78	Pseudo *R* ^2^ = 0.220
GLMM	amt + ants + other.h	1.43	3.93E‐02	−192.95	Pseudo *R* ^2^ = 0.235
GLMM	ants + ant.h + all.h	1.61	3.59E‐02	−191.83	Pseudo *R* ^2^ = 0.251

a The environmental variables included in these models are annual mean temperature (amt), annual mean precipitation (amp), ant mound density per hectare (ants), diversity of nonant arthropods in pitfall traps (other.h), diversity of ants in pitfall traps (ant.h), percentage of ground area that was sage (sage), percentage of ground that was bare soil (soil), survey start time (s.time), and temperature at the start of each survey (s.temp).

b Models were ranked using Akaike's Information Criterion with correction for number of parameters (AICc), which was also used to calculate a weight for each model (AICω).

c Occupancy model fits were assessed using a chi‐square test of observed and bootstrapped data. Models were considered a good fit if the observed test statistic was within the distribution of the bootstrapped test statistics (i.e. *p* ≫ 0.05). Abundance model fits were quantified using coefficient of determination (*R* ^2^) from linear regression results.

The top model for lizard abundance, when zeros were excluded, included linear effects of precipitation, temperature, and ant mound density (Table 1, Table S4). This model showed a significant though somewhat weak fit to the data (*R* ^2^ = .23) and the top supported factors all had substantial effects on predicted abundance (Table S6).

The best linear mixed effects model that we applied to the full dataset (including zeros) included ant mound density plus diversity of all invertebrates (Pseudo‐*R* ^2^ = .24, Table S5); temperature and precipitation were not part of the model. In this case, the signs of the fixed effects (ants and all invertebrates) were the same as in the occupancy and zero‐eliminated abundance models.

**Figure 2:**
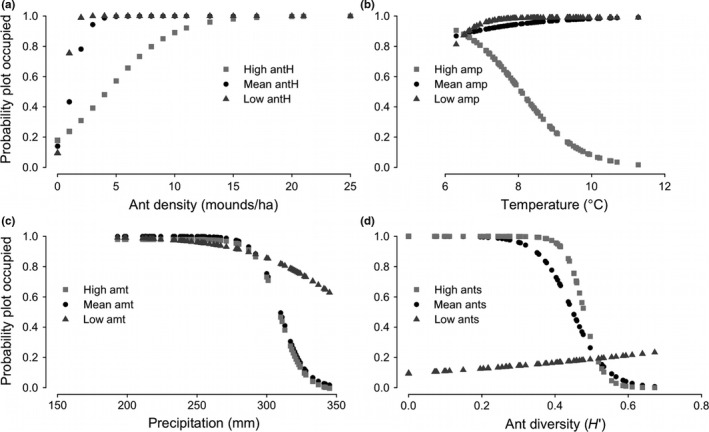
Temperature, precipitation, density of ant mounds, and ant diversity were the environmental factors best supported as influencing probability of horned lizard occupancy. To account for interactions in the top model, relationships are given that hold other interacting variables constant at either mean (black circles), high (dark gray triangles), or low (light gray squares) values. Probability of occupancy is plotted against (a) ant mound density (ants), with the interacting variable of ant diversity (antH) at low (1st quartile), mean, and high (3rd quartile) values. Likewise, probability of occupancy is plotted against b) annual mean temperature (amt) with annual mean precipitation (amp) held at low, mean, and high values and (c) annual mean precipitation, with temperature held at low, mean, and high values. Finally, we plotted (d) the probability of occupancy in response to Shannon diversity (*H*′) of available ants (antH) at low, mean, and high values of ant mound density. Figures presented here without confidence intervals for readability (see Fig. S4 for CIs)

**Figure 3:**
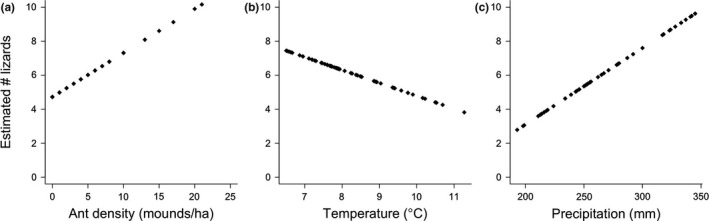
Best environmental predictors of lizard abundance. We report the effect of (a) temperature on predicted lizard abundance at mean precipitation, (b) precipitation on estimated lizard abundance at mean temperature; and across the range of (c) ant mound densities (mounds/ha). Figures presented here without confidence intervals for readability (see Fig. S6 for CIs)

Ant mound density had a strong positive effect on both occupancy and lizard abundance. Predicted occupancy probability increased from 0.49–1 over the range of ant mound densities (Figure 2a, Figure S4, Table S6), while lizard abundance increased from 4.7 to 11.1 lizards over the same range (Figure 3a, Figure S5, Table S6).

In contrast, the relative importance and the combined effect of temperature and precipitation effects had opposing effects in occupancy vs. abundance predictions (Table 2). Temperature had a positive effect on predicted occupancy rates, except where precipitation was high, (increasing probability of occupancy from 0.37 to 0.99; Figure 2b, Figure S4, Table S6), and precipitation had an overall negative effect on occupancy (decreasing probability of occupancy from 0.99–0.37; Figure 2c, Figure S4, Table S6). In plots occupied by lizards, however, temperature negatively affected estimated lizard abundance (decline from 7.4–3.8 lizards; Figure 3b, Fig. S6, Table S6) over the range of observed temperatures; whereas precipitation had a positive effect on predicted lizard abundance (increase from 2.7 – 9.6 lizards; Figure 3c, Table S6). Finally, ant diversity had a negative effect on predicted occupancy rates (decreasing probability of occupancy from 1–0; Figure 1d, Table S6) and no significant effect on abundance. We predicted occupancy and abundance for the three top models (Table S6) and observed similar, although weaker, relationships for lower‐ranked models.

**Table 2:** Support and sign of effect for environmental variables included in occupancy and abundance predictions, excluding covariates for detection; summed AICc weights (AICω) and sign of effect (+,−) are shown for occupancy and abundance model sets, and Italics indicate different signs of effect between occupancy and abundance

Predictor variable	Description	Occupancy AICcω and direction of effect	Estimated abundance AICcω and direction	GLMM AICcω and direction
Annual mean temperature (amt)	Annual mean temperature from iButton data	*0.88 +,* −a	*0.86* −	*0.46* −
Annual mean precipitation (amp)	Annual mean precipitation from WorldClim dataset	*0.88* −	*0.79 +*	*0.17 +*
Ant mound density (ants)	Density of ant mounds—*Formica ravida* and *Pogonomyrmex occidentalis*	0.91 +	0.94 +	0.99 +
Ant *H*′ (ant.h)	Shannon–Wiener diversity of ants collected in pitfall traps	*0.74* −	*0.28* +	*0.39* −
Other invertebrate *H*′ (other.h)	Shannon–Wiener diversity of nonant invertebrate collected in pitfall traps	0.44 −	0.20 −	0.25 −
All *H*′ (all.h)	Shannon–Wiener diversity of all invertebrates collected in pitfall traps	0.17 −	0.23 −	0.59 −
Sage	% of plot with sage (mostly *Artemesia tridentata*) cover	*0.31 +*	*0.24* −	*0.27 +*
Soil	% of plot with soil cover	*0.22 +*	*0.22* −	*0.23* −

a Sign depends on aspects of an interacting variable.

**Figure 4:**
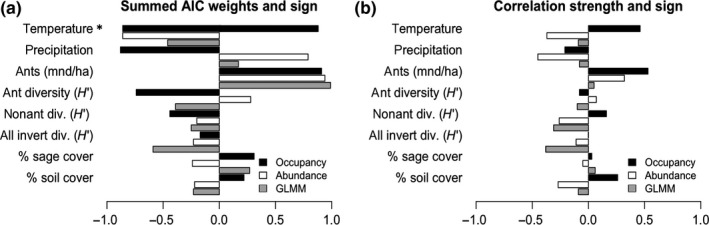
Comparison of the effects of different predictors on lizard occupancy and abundance (with and without zeros included). (a) To identify relative support for individual environmental predictor variables, we added the weights of all models that include that variable. These summed AIC weights (AICω) indicate relative support for each predictor variable within the model set. Here, we compare the top predictors of plot occupancy probabilities and horned lizard abundance using summed AIC weights and direction of effect for occupancy (solid), abundance without zeros (white), and abundance with zeros (GLMM [gray]). (b) Another measure of the effect of each variable on occupancy or abundance is the correlation between each variable and the predicted occupancy or abundance across our study plots. “Temperature” is annual mean temperature and an asterisk indicates that the direction of this effect for occupancy depends on the range of an interacting variable (precipitation), “precipitation” is annual mean precipitation, “ants” is the density of ant mounds per hectare, “ant diversity (*H*′)” is the Shannon diversity of ants in pitfall traps at plots, “nonant div (*H*′)” is the Shannon diversity of nonant invertebrates (e.g. beetles, flies, etc.) in pitfall traps, “all invert div (*H*′)” is the Shannon diversity of all invertebrates in pitfall traps at plots, % sage cover is the estimated percentage of ground covered by *Artemesia* spp., and % soil cover is the estimated percentage of ground covered by bare ground

Summed AIC weights (Figure 4a) were similar between abiotic predictor variables of occupancy and estimated abundance (without zeros), but had few consistencies with the mixed effects model approach (with zeros); sign was inconsistent for precipitation and, in most cases, temperature. Correlation strengths (Figure 4b) were likewise in consistent in both strength and sign. The interactive term of annual mean temperature and precipitation had similarly high summed AIC weights in both approaches (occupancy AICcω = 0.88; abundance AICcω = 0.89), but the effect of temperature changed signs from a positive effect on occupancy to a negative effect on abundance. Similarly, precipitation changed from a negative to positive relationship in each approach. Ant mound density had similar effects (but in the same direction) on occupancy (AICω = 0.91) and abundance (AICω = 0.96) (Table 1).

**Figure 5:**
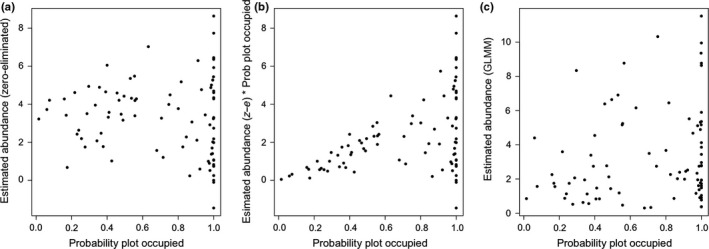
The probabilities of plot occupancy and predicted abundance of lizards were generated from the best models for each approach, for all 89 plots. If the approaches were similar, the relationship between (a) predicted abundance and probability of plot occupancy would be strong and relatively linear, and so would (b) the product of these values (predicted mean abundance corrected for probability of occupancy) plotted against predicted occupancy. Likewise, the (c) predicted abundance generated from the linear mixed models, including zeros, should be correlated with the probability of plot occupancy

Overall, the probability that a plot was occupied did not predict estimated lizard abundance if occupied (*R* ^2^ = 0.4, Figure 5a). While mean abundance corrected for occupancy probability corresponded much more strongly with simple occupancy probability (Figure 5b; *R* ^2^ = .63), there was still substantial variation in predicted abundance above an occupancy probability of 0.4 and even more variation in estimated abundance for sites that had an occupancy probability of approximately one. Probability that a plot was occupied did not predict results from the mixed effects model (*R* ^2^ = .07, Figure 5c).

## 4 DISCUSSION

We found that the environmental factors that best predict site occupancy and abundance can differ substantially. By analyzing our presence–absence data separately from abundance estimates, we observed how differently some environmental factors behaved in predicting occupancy than in predicting abundance. Temperature and precipitation were important components of the best models in the occupancy and zero‐eliminated abundance approaches; precipitation and, in a portion of instances, temperature had opposite relationships with occupancy compared to abundance. When we evaluated presence and abundance data together in the mixed effects model analysis, this complexity was washed out.

Our findings indicate that caution is needed in uniting predictions of occupancy and abundance.

Ant mound density, a measure of prey availability, positively influenced both occupancy and abundance. The best‐supported climatic variables were included in models for both site occupancy and lizard abundance, but the direction and strength of these effects were inconsistent. Greater short‐horned lizards have a broad thermal tolerance (Sherbrooke, 2003), which is likely why lizards were present over much of the range of plot temperatures. Temperature is a well‐supported predictor of occupancy, making our finding of a higher probability of occupancy at higher temperatures unsurprising. The increase in probability of plot occupancy with temperature could be related to temperature‐mediated length of the active season for lizards. While our focal species is viviparous (Sherbrooke, 2003), an adaptation thought to enable reproduction in colder climates (Hodges, 2004), temperature can still affect the development time for lizard embryos (Andrews, Qualls, & Rose, 1997); small differences in annual mean temperatures could affect reproductive success. This relationship broke down at high precipitation values where temperature had a negative affect on occupancy; negative effects of precipitation on occupancy (discussed below) appeared to override temperature effects.

These explanations do not, however, describe why predicted lizard abundance in occupied sites declined at higher temperatures. This decline may be due to the lower temperatures that greater short‐horned lizards prefer compared to other horned lizards (Sherbrooke, 2003). Physiological performance in lizards is often correlated with temperature (Huey & Stevenson, 1979), and it is possible that adaptation to function at lower temperatures limits success at higher temperatures; probability of occupancy may go up but individual and population health decline with rising mean temperatures. Alternatively, lizard abundance may decline with temperature due to other factors correlated with temperature. For example, higher temperatures could indicate the increased abundance of other species, including predatory snakes or competing lizards. Or, higher temperatures could mean higher lizard metabolic rates, with a concomitant higher energetic demand such that the same amount of food then supports fewer lizards.

Horned lizards were less likely to occupy sites with higher precipitation. In plots occupied by lizards, however, lizard abundance increased with precipitation. One potential explanation for lower probability of occupancy is that the summer season could be shorter at these sites due to late snowmelt. Although plots themselves were topographically similar, differences in surrounding topography could affect annual mean precipitation (Gao, Xu, Zhao, Pal, & Giorgi, 2006) in ways that we did not assess. Higher precipitation could improve growth and survival of extant populations. Increased precipitation can increase plant growth and invertebrate abundance, which would mean more prey for horned lizards and, consequently, potentially greater reproductive by lizards.

More broadly, the mismatch we found in predictions of occupancy and abundance may result from the processes governing these two state variables operating at different spatial scales (Orrock et al., 2000). The factors influencing occupancy (perhaps governed by regional population dynamics) are likely to operate at a broader scale than abundance, and this difference may well account for discrepancies between occupancy and abundance approaches (He & Gaston, 2000). Environmental factors may act as coarse filters to dictate where species can and cannot persist, with more local characteristics governing abundance patterns. For example, studies of the reticulate collared lizard indicate that the species is limited to arid, sparsely vegetated thorn brush with sandy soil and rocks for basking (e.g. McGuire, 1996). These characteristics determine where the species can persist, acting as a coarse filter on species distribution across a broad scale. In contrast, abundance of this highly territorial species is likely to be determined more by the availability of territories (e.g. Husak, Lappin, Fox, & Lemos‐Espinal, 2006).

Similar comparisons of environmental factors determining occupancy and abundance are rare. Orrock et al. (2000) found that presence/absence could be useful for predicting habitat quality for a small mammal, but the quality of the prediction depended on the spatial scale of the observation. Like us, Jiménez‐Valverde, Diniz, de Azevedo, and Borges (2009) found poor correlation between presence and abundance across 48 arthropod species. These results, together with our observations, indicate the correspondence between occupancy and abundance is not simple or straightforward and that we stand to learn more from separating occupancy and abundance analyzes than by considering the two together in a single united analysis.

Another possible reason for mismatches between occupancy and abundance patterns comes from theories of range limits and the processes driving these limits. In particular, some range limits may be governed by metapopulation dynamics (Holt & Keitt, 2000), with sparser patches of habitat meaning that occupancy rates decline along an environmental gradient, but without any necessary change in the abundance in suitable sites. A related idea is that fairly simple abiotic effects may set some range limits (geographic or climatic) directly, while other climatic factors or ecological interactions set other range limits (Holt, 2003; Sexton, McIntyre, Angert, & Rice, 2009; Louthan, Doak, & Angert, 2015). In these scenarios, it would not be surprising to see increasing frequency of sites that could support at least some population of a species with, increasing temperature, for example, while at the same time seeing that the same increases result in greater biotic pressures that push densities downwards. Whether these mismatches are common or unusual will remain unclear until we make more direct comparisons between occupancy and abundance.

Our results have direct conservation implications, especially in the face of rapid environmental changes that threaten reptiles (Gibbon et al., 2000). It is important to understand how frequently and seriously the results of occupancy and abundance analyzes diverge in their results. For example, the presence of a species in a landscape of sink and source populations (Donovan & Flather, 2002) is not necessarily indicative of good habitat because environmental characteristics that make patches inhabitable may not have similar effects on population numbers or dynamics. More broadly, even abundance is not necessarily a good indicator of habitat quality, an observation made over 30 years ago by van Horne (1983). While neither occupancy nor abundance analyzes are foolproof approaches to species monitoring, there is conservation value in understanding how environmental factors separately affect the presence of a species and abundance of that species; tying those factors to population fitness would be a more successful approach altogether.

Decoupling occupancy and abundance may be critical to designing effective conservation strategies. In particular, uniting occupancy and abundance analyses could mask the environmental factors that determine occupancy as separate from abundance. An occupancy analysis approach is an advantage when obtaining abundance data are very difficult. While occupancy modeling can be used successfully in estimating abundance, even where detection is heterogeneous (e.g. Royle & Nichols, 2003), the environmental factors that best predict the presence do not necessarily best predict abundance. This caveat may be particularly important for 1) species that are difficult to detect (do not have enough numbers) or where confidence intervals on estimates are very large, and 2) rare species that are widespread (e.g. wolverine; Ellis et al., 2014). Time and monetary constraints, balanced with feasibility and accuracy requirements will often dictate if abundance estimates or presence–absence approach is more appropriate (Joseph, Field, Wilcox, & Possingham, 2006).

Occupancy modeling has gained recent attention for good reasons, but managers should use this approach cautiously. It is important to consider how abundance and occupancy patterns may differ for a species and how those differences could influence the outcome of management strategies based on one analytical approach. We do not suggest that conservation efforts should always be based on estimating only occupancy or abundance, but that the approach be selected carefully to match the objective and with an understanding of the limitations of each. Likewise, to show how common it is for occupancy and abundance to be decoupled, further work on other species is required. Understanding how these factors may lead to differences between results from occupancy and abundance models will help managers use the most efficient and appropriate methods to achieve their conservation goals.

## CONFLICT OF INTEREST

None declared.

## DATA ACCESSIBILITY

Habitat, survey, abundance, and occupancy data are available as text files through ResearchGate: https://www.researchgate.net/profile/Reilly_Dibner/contributions.

## References

[ece33131-bib-0001] Andrews, R. M. , Qualls, C. P. , & Rose, B. R. (1997). Effects of low temperature on embryonic development of Sceloporus lizards. Copeia, 1997, 827.

[ece33131-bib-0003] Beauchamp, B. , Wone, B. , Bros, S. , & Kutilek, M. (1998). Habitat use of the flat‐tailed horned lizard (*Phrynosoma mcallii*) in a disturbed environment. Journal of Herpetology, 32, 210.

[ece33131-bib-0004] Borgelt, A. , & New, T. R. (2006). Pitfall trapping for ants (Hymenoptera, Formicidae) in mesic Australia: What is the best trapping period? Journal of Insect Conservation, 10, 75–77.

[ece33131-bib-0005] Butchart, S. H. M. , Walpole, M. , Collen, B. , van Strien, A. , Scharlemann, J. P. W. , Almond, R. E. A. , … Watson, R. (2010). Global biodiversity: Indicators of recent declines. Science, 328, 1164–1168. 20430971 10.1126/science.1187512

[ece33131-bib-0006] Casner, K. L. , Forister, M. L. , Ram, K. , & Shapiro, A. M. (2014). The utility of repeated presence data as a surrogate for counts: A case study using butterflies. Journal of Insect Conservation, 18, 13–27.

[ece33131-bib-0007] Chao, A. (1989). Estimating population size for sparse data in capture–recapture experiments. Biometrics, 45, 427.

[ece33131-bib-0008] Cingolani, A. M. , Cabido, M. , Gurvich, D. E. , Renison, D. , & Díaz, S. (2007). Filtering processes in the assembly of plant communities: Are species presence and abundance driven by the same traits? Journal of Vegetation Science, 18, 911–920.

[ece33131-bib-0009] Comer, P. , Faber‐Langendoen, D. , Evans, R. , Gawler, S. , Josse, C. , Kittel, G. , … Teague, J. (2003). Ecological systems of the United States: A working classification of U.S. terrestrial systems. Arlington, VA: NatureServe.

[ece33131-bib-0010] Copeland, H. E. , Pocewicz, A. , & Kiesecker, J. M. (2011). Geography of energy development in Western North America: Potential impacts on terrestrial ecosystems In Naugle D. E. (Ed.), Energy development and wildlife conservation in Western North America (pp. 7–22). Washington, DC: Island Press/Center for Resource Economics.

[ece33131-bib-0011] Couturier, T. , Cheylan, M. , Bertolero, A. , Astruc, G. , & Besnard, A. (2013). Estimating abundance and population trends when detection is low and highly variable: A comparison of three methods for the Hermann's tortoise: Three methods for estimating *T. hermanni* abundance. The Journal of Wildlife Management, 77, 454–462.

[ece33131-bib-0012] Dawson, T. P. , Jackson, S. T. , House, J. I. , Prentice, I. C. , & Mace, G. M. (2011). Beyond predictions: Biodiversity conservation in a changing climate. Science, 332, 53–58. 21454781 10.1126/science.1200303

[ece33131-bib-0013] Donovan, T. M. , & Flather, C. H. (2002). Relationships among North American songbird trends, habitat fragmentation, and landscape occupancy. Ecological Applications, 12, 364–374.

[ece33131-bib-0014] Elith, J. , & Leathwick, J. R. (2009). Species distribution models: Ecological explanation and prediction across space and time. Annual Review of Ecology, Evolution, and Systematics, 40, 677–697.

[ece33131-bib-0015] Ellis, M. M. , Ivan, J. S. , & Schwartz, M. K. (2014). Spatially explicit power analyses for occupancy‐based monitoring of wolverine in the U.S. Rocky Mountains: Power in occupancy‐based trend detection. Conservation Biology, 28, 52–62. 24001256 10.1111/cobi.12139

[ece33131-bib-0016] Elston, D. A. , Moss, R. , Boulinier, T. , Arrowsmith, C. , & Lambin, X. (2001). Analysis of aggregation, a worked example: Numbers of ticks on red grouse chicks. Parasitology, 122, 563–569. 11393830 10.1017/s0031182001007740

[ece33131-bib-0017] Fletcher, D. , MacKenzie, D. , & Villouta, E. (2005). Modeling skewed data with many zeros: A simple approach combining ordinary and logistic regression. Environmental and Ecological Statistics, 12, 45–54.

[ece33131-bib-0018] Gao, X. , Xu, Y. , Zhao, Z. , Pal, J. S. , & Giorgi, F. (2006). On the role of resolution and topography in the simulation of East Asia precipitation. Theoretical and Applied Climatology, 86, 173–185.

[ece33131-bib-0019] Gibbon, J. W. , Scott, D. E. , Ryan, T. J. , Buhlmann, K. A. , Tuberville, T. D. , Metts, B. S. , … Winne, C. T. (2000). The global decline of reptiles, déjà vu amphibians. BioScience, 50, 653.

[ece33131-bib-0020] Griffin, T. T. (2003). The effect of pitfall trap size and density on ant capture. Records of the South Australian Museum, 7, 319–325.

[ece33131-bib-0021] Hammerson, G. (2007). Phrynosoma hernandesi. The IUCN Red List of threatened species. e.T64076A12741970.

[ece33131-bib-0022] Harrison, X. A. (2014). Using observation‐level random effects to model overdispersion in count data in ecology and evolution. PeerJ, 2, e616. 25320683 10.7717/peerj.616 PMC4194460

[ece33131-bib-0023] He, F. , & Gaston, K. J. (2000). Occupancy–abundance relationships and sampling scales. Ecography, 23, 503–511.

[ece33131-bib-0024] Hijmans, R. J. , Cameron, S. E. , Parra, J. L. , Jones, P. G. , & Jarvis, A. (2005). Very high resolution interpolated climate surfaces for global land areas. International Journal of Climatology, 25, 1965–1978.

[ece33131-bib-0025] Hodges, W. L. (2004). Evolution of viviparity in horned lizards (Phrynosoma): Testing the cold‐climate hypothesis. Journal of Evolutionary Biology, 17, 1230–1237. 15525408 10.1111/j.1420-9101.2004.00770.x

[ece33131-bib-0026] Holt, R. D. (2003). On the evolutionary ecology of species’ ranges. Evolutionary Ecology Research, 5, 159–178.

[ece33131-bib-0027] Holt, R. D. , & Keitt, T. H. (2000). Alternative causes for range limits: A metapopulation perspective. Ecology Letters, 3, 41–47.

[ece33131-bib-0028] Horne, B. V. (1983). Density as a misleading indicator of habitat quality. The Journal of Wildlife Management, 47, 893.

[ece33131-bib-0029] Huey, R. B. , & Stevenson, R. D. (1979). Integrating thermal physiology and ecology of ectotherms: A discussion of approaches. American Zoologist, 19, 357–366.

[ece33131-bib-0030] Husak, J. F. , Lappin, A. K. , Fox, S. F. , & Lemos‐Espinal, J. A. (2006). Bite‐force performance predicts dominance in male venerable collared lizards (*Crotaphytus antiquus*). Ethology, 112, 572–580.

[ece33131-bib-0031] Jiménez‐Valverde, A. , Diniz, F. , de Azevedo, E. B. , & Borges, P. A. V. (2009). Species distribution models do not account for abundance: The case of arthropods on Terceira Island. Annales Zoologici Fennici, 46, 451–464.

[ece33131-bib-0032] Joseph, L. N. , Field, S. A. , Wilcox, C. , & Possingham, H. P. (2006). Presence–absence versus abundance data for monitoring threatened species. Conservation Biology, 20, 1679–1687. 17181803 10.1111/j.1523-1739.2006.00529.x

[ece33131-bib-0033] Keating, K. A. , Schwartz, C. C. , Haroldson, M. A. , & Moody, D. (2002). Estimating numbers of females with cubs‐of‐the‐year in the Yellowstone grizzly bear population. Ursus, 13, 161–174.

[ece33131-bib-0034] Lincoln, F. C. (1930). Calculating waterfowl abundance on the basis of banding returns. United States Department of Agriculture Circular, 118, 1–4.

[ece33131-bib-0035] Louthan, A. M. , Doak, D. F. , & Angert, A. L. (2015). Where and when do species interactions set range limits? Trends in Ecology & Evolution, 30, 780–792. 26525430 10.1016/j.tree.2015.09.011

[ece33131-bib-0036] MacKenzie, D. I. (Ed.) (2006). Occupancy estimation and modeling: Inferring patterns and dynamics of species. Amsterdam ; Boston, MA: Elsevier.

[ece33131-bib-0037] MacKenzie, D. I. , & Bailey, L. L. (2004). Assessing the fit of site‐occupancy models. Journal of Agricultural, Biological, and Environmental Statistics, 9, 300–318.

[ece33131-bib-0038] MacKenzie, D. , & Hines, J. E. (2014). R interface for program PRESENCE. https://www.mbr-pwrc.usgs.gov/software/presence.html.

[ece33131-bib-0039] MacKenzie, D. I. , Nichols, J. D. , Hines, J. E. , Knutson, M. G. , & Franklin, A. B. (2003). Estimating site occupancy, colonization, and local extinction when a species is detected imperfectly. Ecology, 84, 2200–2207.

[ece33131-bib-0040] MacKenzie, D. I. , Nichols, J. D. , Lachman, G. B. , Droege, S. , Andrew Royle, J. , & Langtimm, C. A. (2002). Estimating site occupancy rates when detection probabilities are less than one. Ecology, 83, 2248–2255.

[ece33131-bib-0041] McGuire, J. A. (1996). Phylogenetic systematics of crotaphytid lizards (Reptilia: Iguania: Crotaphytidae). Bulletin of Carnegie Museum of Natural History, 32, 1–143.

[ece33131-bib-0042] NatureServe (2014). NatureServe explorer: An online encyclopedia of life [web application]. Version 7.1. Arlington, VA: NatureServe.

[ece33131-bib-0043] Naugle, D. E. , Doherty, K. E. , Walker, B. L. , Copeland, H. E. , Holloran, M. J. , & Tack, J. D. (2011). Sage‐grouse and cumulative impacts of energy development In Naugle D. E. (Ed.), Energy development and wildlife conservation in Western North America (pp. 55–70). Washington, DC: Island Press/Center for Resource Economics.

[ece33131-bib-0044] Noon, B. R. , Bailey, L. L. , Sisk, T. D. , & Mckelvey, K. S. (2012). Efficient species‐level monitoring at the landscape scale: *Species‐level monitoring* . Conservation Biology, 26, 432–441. 22594594 10.1111/j.1523-1739.2012.01855.x

[ece33131-bib-0045] Orrock, J. L. , Pagels, J. F. , McShea, W. J. , & Harper, E. K. (2000). Predicting presence and abundance of a small mammal species: The effect of scale and resolution. Ecological Applications, 10, 1356–1366.

[ece33131-bib-0046] Parravicini, V. , Morri, C. , Ciribilli, G. , Montefalcone, M. , Albertelli, G. , & Bianchi, C. N. (2009). Size matters more than method: Visual quadrats vs photography in measuring human impact on Mediterranean rocky reef communities. Estuarine, Coastal and Shelf Science, 81, 359–367.

[ece33131-bib-0047] Pocock, M. J. , Frantz, A. C. , Cowan, D. P. , White, P. C. , & Searle, J. B. (2004). Tapering bias inherent in minimum number alive (MNA) population indices. Journal of Mammalogy, 85, 959–962.

[ece33131-bib-0048] Rissing, S. W. (1981). Prey preferences in the desert horned lizard: Influence of prey foraging method and aggressive behavior. Ecology, 62, 1031.

[ece33131-bib-0049] Royle, J. A. (2006). Site occupancy models with heterogeneous detection probabilities. Biometrics, 62, 97–102. 16542234 10.1111/j.1541-0420.2005.00439.x

[ece33131-bib-0050] Royle, J. A. , & Nichols, J. D. (2003). Estimating abundance from repeated presence–absence data or point counts. Ecology, 84, 777–790.

[ece33131-bib-0051] Sala, O. E. , Chapin, F. S. , Armesto, J. J. , Berlow, E. , Bloomfield, J. , Dirzo, R. , … Wall, D. H. (2000). Biodiversity—global biodiversity scenarios for the year 2100. Science, 287, 1770–1774. 10710299 10.1126/science.287.5459.1770

[ece33131-bib-0052] Sexton, J. P. , McIntyre, P. J. , Angert, A. L. , & Rice, K. J. (2009). Evolution and ecology of species range limits. Annual Review of Ecology, Evolution, and Systematics, 40, 415–436.

[ece33131-bib-0053] Shannon, C. E. (1948). A mathematical theory of communication. Bell System Technical Journal, 27, 379–423.

[ece33131-bib-0054] Sherbrooke, W. C. (2003). Introduction to horned lizards of North America. Berkeley, CA: University of California Press.

[ece33131-bib-0055] Sileshi, G. (2007). A method for estimating insect abundance and patch occupancy with potential applications in large‐scale monitoring programmes. African Entomology, 15, 89–101.

[ece33131-bib-0056] Souther, S. , Tingley, M. W. , Popescu, V. D. , Hayman, D. T. , Ryan, M. E. , Graves, T. A. , … Terrell, K. (2014). Biotic impacts of energy development from shale: Research priorities and knowledge gaps. Frontiers in Ecology and the Environment, 12, 330–338.

[ece33131-bib-0057] Tempel, D. J. , & Gutiérrez, R. J. (2013). Relation between occupancy and abundance for a territorial species, the California spotted owl: Spotted owl occupancy and abundance. Conservation Biology, 27, 1087–1095. 23678946 10.1111/cobi.12074

[ece33131-bib-0058] Walston, L. J. , Cantwell, B. L. , & Krummel, J. R. (2009). Quantifying spatiotemporal changes in a sagebrush ecosystem in relation to energy development. Ecography, 32, 943–952.

[ece33131-bib-0059] Wilson, T. L. , & Schmidt, J. H. (2015). Scale dependence in occupancy models: Implications for estimating bear den distribution and abundance. Ecosphere, 6, art168.

[ece33131-bib-0060] Wyoming Game and Fish Department (2010). Wyoming state wildlife action plan. Cheyenne, WY, USA: Wyoming Game and Fish Department.

