# Supplementary material for: Discrepancies in occupancy and abundance approaches to identifying and protecting habitat for an at‐risk species
Source: Ecol Evol. 2017 Jun 15;7(15):5692–702. doi: 10.1002/ece3.3131 (PMC5655793; doi:10.1002/ece3.3131)
Supplement: Supplementary file 1 [file ECE3-7-5692-s001.docx]

## Supplemental Information

**
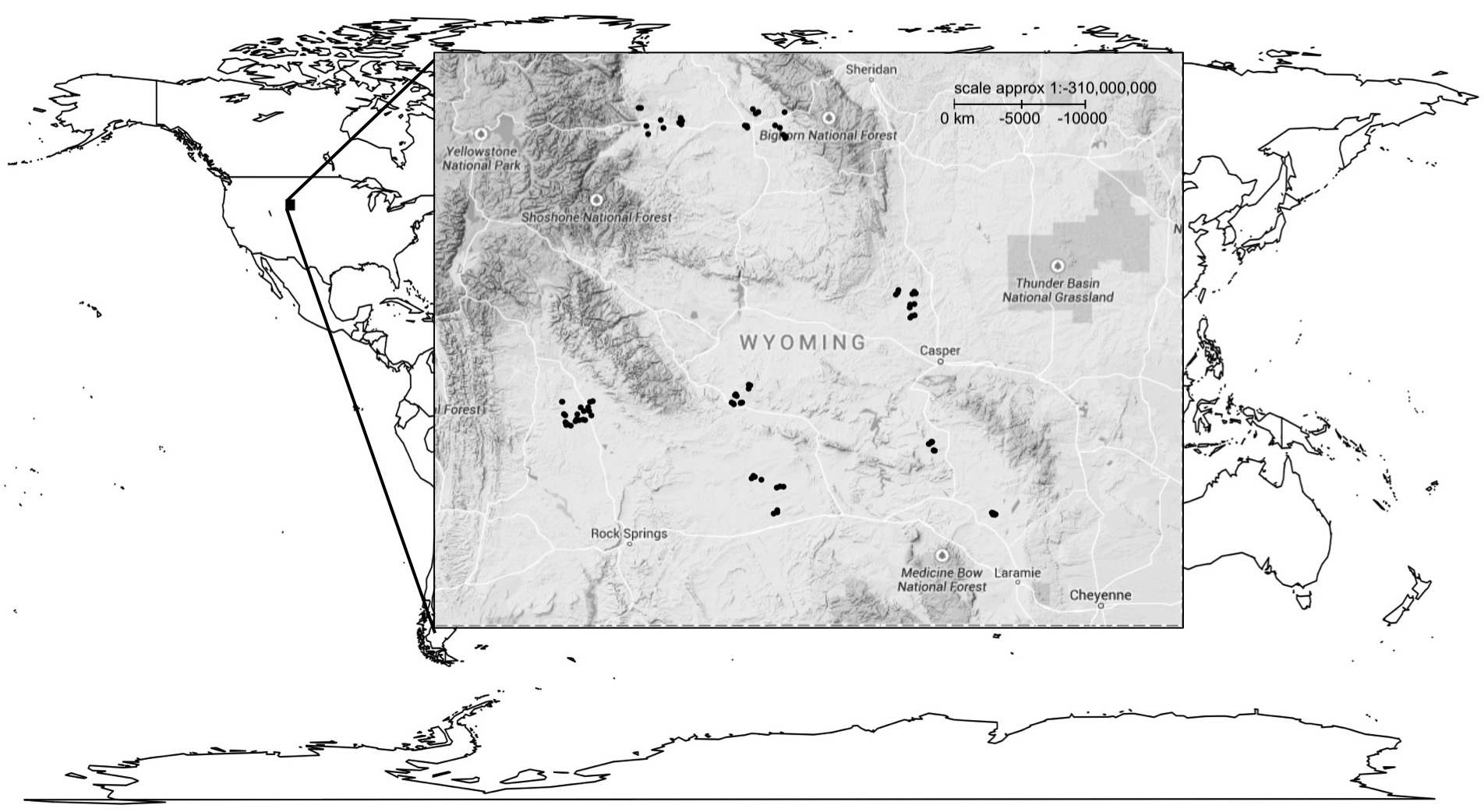
**

**Figure S1.** Locations of the 89 study plots in Wyoming, USA.

**Table S1.** Plot locations for greater short-horned lizard (*Phrynosoma hernandesi*) surveys in 2012 and 2013 in Wyoming, USA.

| Regions | Plot # | Latitude | Longitude | Elevation (m) |
| --- | --- | --- | --- | --- |
| Cody | 1 | 44.536751 | -108.726588 | 1611 |
| Cody | 2 | 44.517149 | -108.712701 | 1552 |
| Cody | 3 | 44.505285 | -108.739679 | 1551 |
| Cody | 4 | 44.492827 | -108.719971 | 1531 |
| Cody | 5 | 44.604297 | -109.095347 | 1654 |
| Cody | 6 | 44.604877 | -109.117047 | 1717 |
| Cody | 7 | 44.482503 | -109.044613 | 1613 |
| Cody | 8 | 44.427771 | -109.029254 | 1664 |
| Cody | 9 | 44.522744 | -108.911335 | 1611 |
| Cody | 10 | 44.469289 | -108.885907 | 1587 |
| Greybull | 1 | 44.470982 | -107.803886 | 1404 |
| Greybull | 2 | 44.486471 | -107.847936 | 1339 |
| Greybull | 3 | 44.485941 | -108.129833 | 1221 |
| Greybull | 4 | 44.483973 | -108.110964 | 1243 |
| Greybull | 5 | 44.471867 | -108.106629 | 1258 |
| Greybull | 6 | 44.411338 | -107.746864 | 1496 |
| Greybull | 7 | 44.400155 | -107.753451 | 1481 |
| Greybull | 8 | 44.423176 | -107.771147 | 1499 |
| Greybull | 9 | 44.577038 | -108.003706 | 1324 |
| Greybull | 10 | 44.566605 | -108.028069 | 1271 |
| Greybull | 11 | 44.596300 | -108.054560 | 1229 |
| Greybull | 12 | 44.575910 | -107.760324 | 1464 |
| Jonah | 1 | 42.502085 | -109.812299 | 2185 |
| Jonah | 2 | 42.588000 | -109.826402 | 2106 |
| Jonah | 3 | 42.588325 | -109.831214 | 2116 |
| Jonah | 8 | 42.592238 | -109.543701 | 2207 |
| Jonah | 9 | 42.586885 | -109.569773 | 2223 |
| Jonah | 11 | 42.492682 | -109.557020 | 2176 |
| Jonah | 12 | 42.445166 | -109.798157 | 2162 |
| Jonah | 13 | 42.493337 | -109.799427 | 2169 |
| Jonah | 18 | 42.432792 | -109.780638 | 2153 |
| Jonah | 20 | 42.548648 | -109.656329 | 2225 |
| Jonah | 21 | 42.500491 | -109.687836 | 2208 |
| Midwest | 1 | 43.188586 | -106.549891 | 1713 |
| Midwest | 2 | 43.184594 | -106.576227 | 1708 |
| Midwest | 3 | 43.172544 | -106.591534 | 1729 |
| Midwest | 4 | 43.261566 | -106.592085 | 1656 |
| Midwest | 5 | 43.264952 | -106.552914 | 1633 |
| Midwest | 6 | 43.240864 | -106.596336 | 1668 |
| Midwest | 7 | 43.338719 | -106.539507 | 1605 |
| Midwest | 8 | 43.351287 | -106.554056 | 1637 |
| Midwest | 9 | 43.335017 | -106.571848 | 1647 |
| Midwest | 10 | 43.330271 | -106.725321 | 1608 |
| Midwest | 11 | 43.343147 | -106.709042 | 1589 |
| Midwest | 12 | 43.361793 | -106.701479 | 1587 |
| Red Desert | 1 | 41.803040 | -107.861411 | 1970 |
| Red Desert | 2 | 41.826826 | -107.828014 | 2079 |
| Red Desert | 3 | 41.812041 | -107.827405 | 2082 |
| Red Desert | 4 | 42.041635 | -107.975597 | 2021 |
| Red Desert | 5 | 42.061872 | -108.036553 | 2005 |
| Red Desert | 6 | 42.068877 | -108.049905 | 2022 |
| Red Desert | 7 | 42.051453 | -108.067109 | 2008 |
| Red Desert | 8 | 41.992756 | -107.770072 | 1998 |
| Red Desert | 9 | 41.994623 | -107.802984 | 2007 |
| Red Desert | 10 | 41.986200 | -107.832634 | 2002 |
| Shirley Basin | 5 | 42.308949 | -106.393119 | 2212 |
| Shirley Basin | 6 | 42.307787 | -106.387279 | 2226 |
| Shirley Basin | 7 | 42.304451 | -106.391238 | 2218 |
| Shirley Basin | 8 | 42.305480 | -106.397375 | 2223 |
| Shirley Basin | 11 | 42.293187 | -106.415078 | 2247 |
| Shirley Basin | 12 | 42.293463 | -106.419853 | 2255 |
| Shirley Basin | 13 | 42.248547 | -106.372317 | 2165 |
| Shirley Basin | 14 | 42.247396 | -106.366578 | 2158 |
| Shirley Basin | 15 | 42.245256 | -106.362849 | 2160 |
| Shirley Basin | 16 | 42.246578 | -106.373371 | 2156 |
| Spring Creek | 1 | 41.794684 | -105.804629 | 2122 |
| Spring Creek | 2 | 41.797665 | -105.797298 | 2115 |
| Spring Creek | 3 | 41.798739 | -105.800616 | 2119 |
| Spring Creek | 4 | 41.799312 | -105.805542 | 2118 |
| Spring Creek | 5 | 41.807017 | -105.818097 | 2118 |
| Spring Creek | 6 | 41.815309 | -105.803866 | 2118 |
| Spring Creek | 7 | 41.807749 | -105.795786 | 2118 |
| Spring Creek | 8 | 41.796328 | -105.823346 | 2110 |
| Spring Creek | 9 | 41.808701 | -105.830270 | 2092 |
| Spring Creek | 10 | 41.807109 | -105.828950 | 2111 |
| Spring Creek | 11 | 41.804664 | -105.828715 | 2105 |
| Spring Creek | 12 | 41.809449 | -105.833017 | 2097 |
| Sweetwater | 1 | 42.679671 | -108.094525 | 2175 |
| Sweetwater | 2 | 42.707319 | -108.092940 | 2179 |
| Sweetwater | 3 | 42.702763 | -108.078932 | 2172 |
| Sweetwater | 4 | 42.634036 | -108.221187 | 2036 |
| Sweetwater | 5 | 42.640554 | -108.213708 | 2070 |
| Sweetwater | 6 | 42.628578 | -108.204859 | 2055 |
| Sweetwater | 7 | 42.582909 | -108.154126 | 2027 |
| Sweetwater | 8 | 42.579065 | -108.168436 | 2018 |
| Sweetwater | 9 | 42.582261 | -108.165727 | 2017 |
| Sweetwater | 10 | 42.582927 | -108.252823 | 2041 |
| Sweetwater | 11 | 42.576066 | -108.235939 | 2033 |
| Sweetwater | 12 | 42.570429 | -108.231578 | 2017 |

**Table S2.** Summary of results for detection models on a constant (1) probability of plot occupancy (ψ). Detection models (ρ) included a null (constant) model and various combinations of the following variables: start temperature of survey (s.temp), start time of survey (s.time), finish temperature of survey (f.temp), finish time of survey (f.time), sagebrush cover percentage (sage), soil cover percentage (soil), and grass cover percentage (grass).

| Ψ structure | ρ structure | AICc | ΔAIC | modlike | AICω | Detection probability |
| --- | --- | --- | --- | --- | --- | --- |
| 1 | s.temp | 134.27 | 0.00 | 0.261 | 0.23 | 0.89 |
| 1 | 1 | 135.93 | 1.66 | 0.114 | 0.12 | 0.90 |
| 1 | sage | 135.98 | 1.70 | 0.111 | 0.17 | 0.91 |
| 1 | soil | 136.15 | 1.88 | 0.102 | 0.11 | 0.93 |
| 1 | s.time | 136.27 | 2.00 | 0.096 | 0.10 | 0.87 |
| 1 | f.time * f.temp | 136.27 | 2.00 | 0.096 | 0.10 | 0.84 |
| 1 | s.time * s.temp | 137.81 | 3.53 | 0.045 | 0.04 | 0.83 |
| 1 | grass + f.time * f.temp | 137.93 | 3.65 | 0.042 | 0.04 | 0.91 |
| 1 | grass | 137.93 | 3.65 | 0.042 | 0.04 | 0.91 |
| 1 | sage + f.time * f.temp | 138.17 | 390 | 0.037 | 0.04 | 0.84 |
| 1 | soil + f.time * f.temp | 138.26 | 3.99 | 0.035 | 0.04 | 0.84 |
| 1 | s.time * s.temp + f.temp | 139.80 | 5.53 | 0.016 | 0.02 | 0.89 |

**Comparison of Detection Probabilities in Occupancy Models**

Different combinations of site and survey-specific variables gave detection probabilities ranging from 0.83 (± 0.09 SE; Appendix S2) to 0.93 (± 0.06 SE) across all plots. The best model for ρ with constant occupancy (ψ = 1) had a detection probability of 0.89 (± 0.06 SE). Parallel model runs for the two next best models produced the same results (Appendix S3).

The best occupancy model on detection covariates for the best detection model had mean site occupancy of 0.72 (± 0.12 SE). Occupancy estimates were insensitive to the differences in detection probabilities, with substantial overlap in standard error. The top models based on the best covariates predicting occupancy without habitat covariates were similar to the models using covariates that gave the highest detection probability.

**Table S3.** Summary of results for 320 occupancy models run on the best detection model (s.temp), where Ψ structure is the model for occupancy. Occupancy models included the following variables: annual mean temperature (amt), annual mean precipitation (amp), elevation (elev), ant mound density per hectare (ants), the Shannon diversity of available ants (antH), the Shannon diversity of available non-ant invertebrates (otherH), the Shannon diversity of all available invertebrates (allH), the percentage of ground covered by sage (sage), and the percentage of ground covered by soil (soil).

| Ψ structure^a^ | AICc | ΔAIC | modlike | AICω |
| --- | --- | --- | --- | --- |
| amt * amp + ants * ant.h | 114.47 | 0 | 1.00 | 9.41E-02 |
| amt + amp + ants * ant.h | 115.58 | 1.11 | 0.57 | 5.40E-02 |
| amt + amp + ants * ant.h + other.h + sage | 115.69 | 1.22 | 0.54 | 5.12E-02 |
| amt * amp + ants * ant.h + other.h | 115.76 | 1.29 | 0.52 | 4.94E-02 |
| amt + amp + ants * ant.h + other.h + soil | 115.83 | 1.36 | 0.51 | 4.76E-02 |
| amt + amp + ants + soil | 116.37 | 1.90 | 0.39 | 3.64E-02 |
| amt * amp + ants * ant.h + sage | 116.39 | 1.92 | 0.38 | 3.60E-02 |
| amt * amp + ants * ant.h + other.h + all.h | 116.80 | 2.33 | 0.31 | 2.93E-02 |
| amt * amp + ants | 117.37 | 2.90 | 0.23 | 2.21E-02 |
| amt + amp + ants * ant.h + all.h | 117.46 | 2.99 | 0.22 | 2.11E-02 |
| amt + amp + ants * ant.h + sage | 117.48 | 3.01 | 0.22 | 2.09E-02 |
| amt + amp + ants * ant.h + other.h | 117.56 | 3.09 | 0.21 | 2.01E-02 |
| amt * amp + ants * ant.h + other.h + sage | 117.77 | 3.30 | 0.19 | 1.80E-02 |
| amt * amp + ants * ant.h + other.h + soil | 117.78 | 3.31 | 0.19 | 1.80E-02 |
| amt + amp + ants | 117.81 | 3.34 | 0.19 | 1.77E-02 |
| amt * amp + ants + soil | 118.17 | 3.70 | 0.16 | 1.48E-02 |
| amt + amp + ants + all.h + soil | 118.30 | 3.83 | 0.15 | 1.39E-02 |
| amt + amp + ants + ant.h + soil | 118.32 | 3.85 | 0.15 | 1.37E-02 |
| amt + amp + ants + other.h + soil | 118.48 | 4.01 | 0.13 | 1.27E-02 |
| amt * amp + ants * ant.h + other.h + all.h + sage | 118.78 | 4.31 | 0.12 | 1.09E-02 |
| amt * amp + ants + all.h | 118.82 | 4.35 | 0.11 | 1.07E-02 |
| amt * amp + ants * ant.h + other.h + sage + soil | 119.15 | 4.68 | 0.10 | 9.06E-03 |
| amt * amp + ants + ant.h | 119.20 | 4.73 | 0.09 | 8.86E-03 |
| amt * amp + ants + sage | 119.21 | 4.74 | 0.09 | 8.79E-03 |
| amt + amp + ants * ant.h + soil | 119.29 | 4.82 | 0.09 | 8.46E-03 |
| amt * amp + ants + other.h | 119.38 | 4.91 | 0.09 | 8.07E-03 |
| amt + amp + ants * ant.h + all.h + sage | 119.43 | 4.96 | 0.08 | 7.90E-03 |
| amt + amp + ants + sage | 119.59 | 5.12 | 0.08 | 7.28E-03 |
| amt * amp + ants + all.h + soil | 119.62 | 5.15 | 0.08 | 7.16E-03 |
| amt + amp + ants + all.h | 119.77 | 5.30 | 0.07 | 6.65E-03 |
| amt + amp + ants + ant.h | 119.78 | 5.31 | 0.07 | 6.61E-03 |
| amt + amp + ants + other.h | 119.78 | 5.31 | 0.07 | 6.60E-03 |
| ants * ant.h | 119.84 | 5.37 | 0.07 | 6.42E-03 |
| amt * amp + other.h | 119.88 | 5.41 | 0.07 | 6.29E-03 |
| amt + amp + ants + other.h + all.h + soil | 119.94 | 5.47 | 0.06 | 6.09E-03 |
| amt + amp + ants + other.h + all.h + soil | 119.94 | 5.47 | 0.06 | 6.09E-03 |
| amt + amp + ants + ant.h + all.h + soil | 120.00 | 5.53 | 0.06 | 5.92E-03 |
| amt * amp + ants + ant.h + soil | 120.03 | 5.56 | 0.06 | 5.85E-03 |
| amt + amp + ants + other.h + sage + soil | 120.10 | 5.63 | 0.06 | 5.63E-03 |
| amt * amp + ants + other.h + soil | 120.16 | 5.69 | 0.06 | 5.47E-03 |
| amt + amp + ants + ant.h + sage + soil | 120.21 | 5.74 | 0.06 | 5.34E-03 |
| amt * amp + ants + sage + soil | 120.24 | 5.77 | 0.06 | 5.26E-03 |
| ants * ant.h + other.h + all.h | 120.45 | 5.98 | 0.05 | 4.72E-03 |
| amt + amp + ants * ant.h + other.h + all.h | 120.48 | 6.01 | 0.05 | 4.66E-03 |
| amt * amp + ants + ant.h + other.h + all.h | 120.53 | 6.06 | 0.05 | 4.54E-03 |
| amt * amp + ants + other.h + sage + soil | 120.63 | 6.16 | 0.05 | 4.33E-03 |
| amt * amp + ants + all.h + sage | 120.65 | 6.18 | 0.05 | 4.29E-03 |
| amt * amp + ants + other.h + all.h | 120.70 | 6.23 | 0.04 | 4.17E-03 |
| ants * ant.h + sage | 120.81 | 6.34 | 0.04 | 3.95E-03 |
| amt * amp + ants + ant.h + all.h | 120.81 | 6.34 | 0.04 | 3.95E-03 |
| ants * ant.h + other.h | 120.93 | 6.46 | 0.04 | 3.72E-03 |
| amt + amp + ants * ant.h + sage + soil | 120.96 | 6.49 | 0.04 | 3.67E-03 |
| amt * amp + ants + ant.h + other.h | 120.99 | 6.52 | 0.04 | 3.62E-03 |
| amt * amp + ants + ant.h + sage | 121.03 | 6.56 | 0.04 | 3.55E-03 |
| amt * amp + other.h + soil | 121.06 | 6.59 | 0.04 | 3.49E-03 |
| ants | 121.09 | 6.62 | 0.04 | 3.44E-03 |
| amt + amp + ants * ant.h + all.h + soil | 121.13 | 6.66 | 0.04 | 3.37E-03 |
| amt * amp + ants + ant.h + other.h + all.h + sage + soil | 121.13 | 6.66 | 0.04 | 3.36E-03 |
| amt + amp + ants + ant.h + other.h + all.h + soil | 121.22 | 6.75 | 0.03 | 3.22E-03 |
| amt * amp + ants + other.h + sage | 121.30 | 6.83 | 0.03 | 3.09E-03 |
| amt * amp + other.h + sage | 121.41 | 6.94 | 0.03 | 2.93E-03 |
| amt + ants * ant.h | 121.43 | 6.96 | 0.03 | 2.90E-03 |
| amt + amp + ants + other.h + sage | 121.52 | 7.05 | 0.03 | 2.77E-03 |
| amt * amp | 121.56 | 7.09 | 0.03 | 2.72E-03 |
| amt + amp + ants + ant.h + sage | 121.58 | 7.11 | 0.03 | 2.70E-03 |
| amt + amp + ants + all.h + sage | 121.58 | 7.11 | 0.03 | 2.69E-03 |
| amt * amp + other.h + all.h | 121.60 | 7.13 | 0.03 | 2.67E-03 |
| amt * amp + ants + ant.h + all.h + soil | 121.60 | 7.13 | 0.03 | 2.66E-03 |
| ants * ant.h + other.h + all.h + sage | 121.61 | 7.14 | 0.03 | 2.66E-03 |
| amt + amp + ants + ant.h + all.h | 121.61 | 7.14 | 0.03 | 2.65E-03 |
| ants * ant.h + all.h | 121.63 | 7.16 | 0.03 | 2.63E-03 |
| ants * ant.h + soil | 121.65 | 7.18 | 0.03 | 2.60E-03 |
| amt + amp + ants + other.h + all.h | 121.71 | 7.24 | 0.03 | 2.52E-03 |
| amt + amp + ants + ant.h + other.h | 121.76 | 7.29 | 0.03 | 2.46E-03 |
| amt + amp + ants + other.h + all.h + sage + soil | 121.80 | 7.33 | 0.03 | 2.42E-03 |
| amt * amp + ant.h + other.h | 121.85 | 7.38 | 0.02 | 2.35E-03 |
| amt * amp + all.h | 121.93 | 7.46 | 0.02 | 2.26E-03 |
| amt * amp + ants + other.h + all.h + soil | 121.94 | 7.47 | 0.02 | 2.24E-03 |
| amt * amp + ants * ant.h + all.h + sage | 122.00 | 7.53 | 0.02 | 2.18E-03 |
| amt * amp + ants + all.h + sage + soil | 122.01 | 7.54 | 0.02 | 2.17E-03 |
| amt + amp + ants + ant.h + other.h + sage + soil | 122.05 | 7.58 | 0.02 | 2.13E-03 |
| ants * ant.h + all.h + sage | 122.09 | 7.62 | 0.02 | 2.08E-03 |
| amt + amp + ants * ant.h + other.h + all.h + soil | 122.10 | 7.63 | 0.02 | 2.08E-03 |
| amt * amp + ants + ant.h + other.h + soil | 122.11 | 7.64 | 0.02 | 2.07E-03 |
| amt * amp + ants + ant.h + sage + soil | 122.20 | 7.73 | 0.02 | 1.97E-03 |
| amt + amp + ants * ant.h + other.h + all.h + sage | 122.30 | 7.83 | 0.02 | 1.88E-03 |
| amt * amp + soil | 122.30 | 7.83 | 0.02 | 1.88E-03 |
| amt * amp + ants * ant.h + soil | 122.31 | 7.84 | 0.02 | 1.87E-03 |
| amt + ants * ant.h + sage | 122.34 | 7.87 | 0.02 | 1.84E-03 |
| amt * amp + sage | 122.35 | 7.88 | 0.02 | 1.83E-03 |
| ants * ant.h + other.h + all.h + soil | 122.35 | 7.88 | 0.02 | 1.83E-03 |
| ants * ant.h + other.h + sage | 122.35 | 7.88 | 0.02 | 1.83E-03 |
| amt + ants * ant.h + other.h + all.h | 122.39 | 7.92 | 0.02 | 1.79E-03 |
| amt * amp + ants + ant.h + other.h + sage + soil | 122.47 | 8.00 | 0.02 | 1.73E-03 |
| ants + sage | 122.52 | 8.05 | 0.02 | 1.68E-03 |
| amt * amp + ants + other.h + all.h + sage | 122.58 | 8.11 | 0.02 | 1.63E-03 |
| amt * amp + ants + ant.h + all.h + sage | 122.59 | 8.12 | 0.02 | 1.63E-03 |
| amt * amp + ant.h + all.h | 122.60 | 8.13 | 0.02 | 1.62E-03 |
| amt + amp + ant.h + other.h + all.h + sage | 122.61 | 8.14 | 0.02 | 1.60E-03 |
| amt + amp + ants * ant.h + other.h + sage + soil | 122.61 | 8.14 | 0.02 | 1.60E-03 |
| ants * ant.h + sage + soil | 122.71 | 8.24 | 0.02 | 1.53E-03 |
| amt + amp + ants + ant.h + other.h + all.h + sage + soil | 122.79 | 8.32 | 0.02 | 1.47E-03 |
| amt * amp + ants * ant.h + all.h | 122.81 | 8.34 | 0.02 | 1.45E-03 |
| amt * amp + other.h + all.h + soil | 122.81 | 8.34 | 0.02 | 1.45E-03 |
| ants * ant.h + other.h + soil | 122.87 | 8.40 | 0.01 | 1.41E-03 |
| amt * amp + all.h + soil | 122.88 | 8.41 | 0.01 | 1.41E-03 |
| amt + ants * ant.h + other.h | 122.89 | 8.42 | 0.01 | 1.40E-03 |
| amt + amp + ants * ant.h + all.h + sage + soil | 122.92 | 8.45 | 0.01 | 1.38E-03 |
| ants + all.h + sage | 122.93 | 8.46 | 0.01 | 1.37E-03 |
| amt * amp + other.h + sage + soil | 122.94 | 8.47 | 0.01 | 1.36E-03 |
| ants + soil | 123.01 | 8.54 | 0.01 | 1.31E-03 |
| amt * amp + ant.h + other.h + soil | 123.04 | 8.56 | 0.01 | 1.30E-03 |
| ants + ant.h | 123.06 | 8.59 | 0.01 | 1.28E-03 |
| amt * amp + ants + ant.h + other.h + sage | 123.12 | 8.65 | 0.01 | 1.24E-03 |
| amt + ants * ant.h + all.h | 123.13 | 8.66 | 0.01 | 1.24E-03 |
| amt * amp + ants * ant.h + other.h + all.h + sage + soil | 123.13 | 8.66 | 0.01 | 1.24E-03 |
| ants + other.h | 123.14 | 8.67 | 0.01 | 1.23E-03 |
| ants + sage + soil | 123.20 | 8.73 | 0.01 | 1.20E-03 |
| amt * amp + ants + ant.h + other.h + all.h + soil | 123.22 | 8.75 | 0.01 | 1.19E-03 |
| amt * amp + all.h + sage | 123.22 | 8.75 | 0.01 | 1.18E-03 |
| amt * amp + other.h + all.h + sage | 123.25 | 8.78 | 0.01 | 1.17E-03 |
| amt + ants * ant.h + soil | 123.34 | 8.87 | 0.01 | 1.12E-03 |
| ants * ant.h + all.h + soil | 123.36 | 8.89 | 0.01 | 1.10E-03 |
| amt + ants | 123.37 | 8.89 | 0.01 | 1.10E-03 |
| amt * amp + ant.h + other.h + sage | 123.37 | 8.90 | 0.01 | 1.10E-03 |
| amt * amp + ants + ant.h + all.h + sage + soil | 123.48 | 9.01 | 0.01 | 1.04E-03 |
| amt * amp + ant.h + other.h + all.h | 123.49 | 9.02 | 0.01 | 1.04E-03 |
| amt + ants * ant.h + all.h + sage | 123.49 | 9.02 | 0.01 | 1.03E-03 |
| ants + all.h | 123.50 | 9.03 | 0.01 | 1.03E-03 |
| amt + amp + ants + ant.h + all.h + sage | 123.50 | 9.03 | 0.01 | 1.03E-03 |
| amt + amp + ants + ant.h + other.h + sage | 123.51 | 9.04 | 0.01 | 1.03E-03 |
| amt * amp + ant.h | 123.56 | 9.09 | 0.01 | 1.00E-03 |
| ants * ant.h + other.h + all.h + sage + soil | 123.60 | 9.13 | 0.01 | 9.81E-04 |
| amt + ants * ant.h + other.h + all.h + sage | 123.60 | 9.13 | 0.01 | 9.79E-04 |
| amt * amp + ants + other.h + all.h + sage + soil | 123.62 | 9.15 | 0.01 | 9.71E-04 |
| amt * amp + ant.h + all.h + soil | 123.71 | 9.24 | 0.01 | 9.27E-04 |
| ants + ant.h + sage | 123.75 | 9.28 | 0.01 | 9.10E-04 |
| ants * ant.h + all.h + sage + soil | 123.81 | 9.34 | 0.01 | 8.81E-04 |
| amt * amp + sage + soil | 123.84 | 9.37 | 0.01 | 8.68E-04 |
| amt + ants * ant.h + sage + soil | 123.84 | 9.37 | 0.01 | 8.68E-04 |
| amt + amp + ants * ant.h + other.h + all.h + sage + soil | 123.86 | 9.39 | 0.01 | 8.62E-04 |
| amt * amp + ants * ant.h + all.h + soil | 123.96 | 9.49 | 0.01 | 8.18E-04 |
| amt + ants + sage | 124.04 | 9.57 | 0.01 | 7.87E-04 |
| amt + ants + sage + soil | 124.14 | 9.67 | 0.01 | 7.48E-04 |
| amt + ants + all.h | 124.14 | 9.67 | 0.01 | 7.48E-04 |
| amt + ants * ant.h + other.h + sage | 124.20 | 9.72 | 0.01 | 7.28E-04 |
| amt * amp + ants * ant.h + sage + soil | 124.20 | 9.73 | 0.01 | 7.26E-04 |
| ants + other.h + sage | 124.27 | 9.80 | 0.01 | 7.00E-04 |
| amt + ants * ant.h + other.h + all.h + soil | 124.27 | 9.80 | 0.01 | 7.00E-04 |
| amt * amp + ant.h + soil | 124.30 | 9.83 | 0.01 | 6.91E-04 |
| amt + ants + all.h + sage | 124.31 | 9.84 | 0.01 | 6.88E-04 |
| amt * amp + ant.h + sage | 124.34 | 9.87 | 0.01 | 6.75E-04 |
| ants * ant.h + other.h + sage + soil | 124.35 | 9.88 | 0.01 | 6.73E-04 |
| amt * amp + ant.h + all.h + sage | 124.36 | 9.89 | 0.01 | 6.71E-04 |
| ants + ant.h + all.h + sage | 124.37 | 9.90 | 0.01 | 6.68E-04 |
| amt * amp + ants + ant.h + other.h + all.h + sage | 124.37 | 9.90 | 0.01 | 6.65E-04 |
| ants + other.h + all.h | 124.39 | 9.92 | 0.01 | 6.60E-04 |
| ants + all.h + sage + soil | 124.41 | 9.94 | 0.01 | 6.52E-04 |
| ants + ant.h + all.h | 124.42 | 9.94 | 0.01 | 6.52E-04 |
| ants + all.h + soil | 124.48 | 10.01 | 0.01 | 6.32E-04 |
| amt + amp + ants + sage + soil | 124.52 | 10.05 | 0.01 | 6.20E-04 |
| amt + amp + ants + ant.h + other.h + all.h | 124.54 | 10.07 | 0.01 | 6.12E-04 |
| amt * amp + all.h + sage + soil | 124.67 | 10.20 | 0.01 | 5.73E-04 |
| amt + ants * ant.h + all.h + sage + soil | 124.68 | 10.21 | 0.01 | 5.72E-04 |
| amt + amp + ants + ant.h + other.h + soil | 124.71 | 10.24 | 0.01 | 5.63E-04 |
| amt * amp + ant.h + other.h + all.h + soil | 124.72 | 10.25 | 0.01 | 5.61E-04 |
| amt * amp + other.h + all.h + sage + soil | 124.75 | 10.28 | 0.01 | 5.51E-04 |
| ants + ant.h + other.h + all.h | 124.83 | 10.36 | 0.01 | 5.28E-04 |
| amt + ants * ant.h + other.h + soil | 124.84 | 10.37 | 0.01 | 5.27E-04 |
| amt * amp + ant.h + other.h + sage + soil | 124.91 | 10.44 | 0.01 | 5.09E-04 |
| ants + ant.h + other.h | 124.96 | 10.49 | 0.01 | 4.97E-04 |
| ants + ant.h + soil | 124.98 | 10.51 | 0.01 | 4.91E-04 |
| amt + ants * ant.h + all.h + soil | 124.99 | 10.52 | 0.01 | 4.90E-04 |
| amt + amp + ants + other.h + all.h + sage | 125.06 | 10.59 | 0.01 | 4.73E-04 |
| amt + ants + other.h | 125.07 | 10.60 | 0.00 | 4.69E-04 |
| ants + other.h + soil | 125.14 | 10.67 | 0.00 | 4.55E-04 |
| amt + ants + all.h + sage + soil | 125.16 | 10.69 | 0.00 | 4.50E-04 |
| ants + other.h + sage + soil | 125.17 | 10.70 | 0.00 | 4.47E-04 |
| ants + ant.h + sage + soil | 125.19 | 10.72 | 0.00 | 4.42E-04 |
| amt * amp + ant.h + other.h + all.h + sage | 125.22 | 10.75 | 0.00 | 4.37E-04 |
| amt * amp + ants * ant.h + other.h + all.h + soil | 125.22 | 10.75 | 0.00 | 4.37E-04 |
| amt + amp + ants + ant.h + other.h + all.h + sage | 125.27 | 10.80 | 0.00 | 4.25E-04 |
| amt + ants + ant.h | 125.36 | 10.89 | 0.00 | 4.06E-04 |
| amt + ants + soil | 125.36 | 10.89 | 0.00 | 4.06E-04 |
| ants + ant.h + other.h + all.h + sage | 125.43 | 10.96 | 0.00 | 3.93E-04 |
| amt * amp + ants * ant.h + all.h + sage + soil | 125.48 | 11.01 | 0.00 | 3.83E-04 |
| ants + other.h + all.h + sage | 125.57 | 11.10 | 0.00 | 3.65E-04 |
| amt + ants * ant.h + other.h + all.h + sage + soil | 125.59 | 11.12 | 0.00 | 3.62E-04 |
| amt * amp + ant.h + all.h + sage + soil | 125.70 | 11.23 | 0.00 | 3.42E-04 |
| ants + ant.h + all.h + sage + soil | 125.71 | 11.24 | 0.00 | 3.40E-04 |
| amt + ants + ant.h + all.h + sage | 125.75 | 11.28 | 0.00 | 3.35E-04 |
| amt + ants * ant.h + other.h + sage + soil | 125.80 | 11.33 | 0.00 | 3.26E-04 |
| ants + ant.h + other.h + all.h + sage + soil | 125.82 | 11.35 | 0.00 | 3.23E-04 |
| amt * amp + ant.h + sage + soil | 125.84 | 11.37 | 0.00 | 3.20E-04 |
| amt + ants + other.h + sage | 126.03 | 11.56 | 0.00 | 2.91E-04 |
| ants + other.h + all.h + sage + soil | 126.03 | 11.56 | 0.00 | 2.91E-04 |
| amt + amp + other.h | 126.03 | 11.56 | 0.00 | 2.91E-04 |
| amt + ants + ant.h + sage | 126.03 | 11.56 | 0.00 | 2.91E-04 |
| ants + ant.h + other.h + sage | 126.08 | 11.61 | 0.00 | 2.83E-04 |
| amt + ants + other.h + sage + soil | 126.09 | 11.62 | 0.00 | 2.82E-04 |
| amt + ants + ant.h + sage + soil | 126.14 | 11.67 | 0.00 | 2.75E-04 |
| amt + all.h + sage + soil | 126.25 | 11.78 | 0.00 | 2.60E-04 |
| amt + other.h + sage + soil | 126.25 | 11.78 | 0.00 | 2.60E-04 |
| amt + ants + other.h + all.h + sage | 126.30 | 11.83 | 0.00 | 2.53E-04 |
| amt + ants + other.h + all.h | 126.33 | 11.86 | 0.00 | 2.50E-04 |
| ants + ant.h + all.h + soil | 126.38 | 11.91 | 0.00 | 2.44E-04 |
| ants + other.h + all.h + soil | 126.39 | 11.92 | 0.00 | 2.43E-04 |
| amt + other.h + all.h + sage | 126.44 | 11.97 | 0.00 | 2.37E-04 |
| amt + amp + ants + all.h + sage + soil | 126.51 | 12.04 | 0.00 | 2.28E-04 |
| amt + ants + ant.h + all.h + sage + soil | 126.54 | 12.07 | 0.00 | 2.25E-04 |
| amt + ants + ant.h + soil | 126.55 | 12.08 | 0.00 | 2.24E-04 |
| amt + ants + other.h + soil | 126.56 | 12.09 | 0.00 | 2.23E-04 |
| amt * amp + ant.h + other.h + all.h + sage + soil | 126.69 | 12.22 | 0.00 | 2.09E-04 |
| amt + ants + ant.h + other.h + all.h | 126.73 | 12.26 | 0.00 | 2.05E-04 |
| ants + ant.h + other.h + soil | 126.78 | 12.31 | 0.00 | 2.00E-04 |
| amt + amp + other.h + all.h | 126.82 | 12.35 | 0.00 | 1.95E-04 |
| ants + ant.h + other.h + all.h + soil | 126.83 | 12.36 | 0.00 | 1.95E-04 |
| amt + ants + ant.h + all.h | 126.96 | 12.49 | 0.00 | 1.82E-04 |
| amt + ants + ant.h + other.h | 127.05 | 12.58 | 0.00 | 1.74E-04 |
| amt + ants + all.h + soil | 127.07 | 12.60 | 0.00 | 1.73E-04 |
| amt + ants + ant.h + other.h + sage | 127.13 | 12.66 | 0.00 | 1.68E-04 |
| amt + ants + other.h + all.h + sage + soil | 127.13 | 12.66 | 0.00 | 1.68E-04 |
| ants + ant.h + other.h + sage + soil | 127.15 | 12.68 | 0.00 | 1.66E-04 |
| amt + amp + all.h | 127.37 | 12.90 | 0.00 | 1.49E-04 |
| amt + ant.h + sage | 127.45 | 12.98 | 0.00 | 1.43E-04 |
| amt + amp + other.h + soil | 127.48 | 13.01 | 0.00 | 1.40E-04 |
| amt + ants + other.h + all.h + soil | 127.51 | 13.04 | 0.00 | 1.39E-04 |
| all.h + sage + soil | 127.56 | 13.09 | 0.00 | 1.35E-04 |
| amt + ants + ant.h + other.h + all.h + sage | 127.62 | 13.15 | 0.00 | 1.31E-04 |
| amt + amp + ant.h + other.h | 127.63 | 13.16 | 0.00 | 1.31E-04 |
| amt + ants + ant.h + other.h + all.h + sage + soil | 127.75 | 13.28 | 0.00 | 1.23E-04 |
| amt + amp + other.h + sage | 127.92 | 13.45 | 0.00 | 1.13E-04 |
| amt + amp | 128.03 | 13.56 | 0.00 | 1.07E-04 |
| amt + amp | 128.03 | 13.56 | 0.00 | 1.07E-04 |
| amt + ants + ant.h + other.h + sage + soil | 128.08 | 13.61 | 0.00 | 1.04E-04 |
| amt + amp + ant.h + all.h | 128.15 | 13.68 | 0.00 | 1.01E-04 |
| amt + ant.h + all.h + sage + soil | 128.25 | 13.78 | 0.00 | 9.58E-05 |
| amt + ant.h + other.h + sage + soil | 128.25 | 13.78 | 0.00 | 9.58E-05 |
| amt + other.h + all.h + sage + soil | 128.25 | 13.78 | 0.00 | 9.58E-05 |
| amt + amp + all.h + soil | 128.43 | 13.96 | 0.00 | 8.75E-05 |
| amt + ant.h + other.h + all.h + sage | 128.44 | 13.97 | 0.00 | 8.71E-05 |
| amt + amp + other.h + all.h + soil | 128.45 | 13.98 | 0.00 | 8.67E-05 |
| amt + amp + ants + ant.h + all.h + sage + soil | 128.51 | 14.04 | 0.00 | 8.42E-05 |
| amt + ants + ant.h + other.h + soil | 128.52 | 14.05 | 0.00 | 8.39E-05 |
| amt + amp + soil | 128.57 | 14.10 | 0.00 | 8.15E-05 |
| amt + amp + ant.h + other.h + all.h | 128.59 | 14.12 | 0.00 | 8.10E-05 |
| amt + ants + ant.h + other.h + all.h + soil | 128.73 | 14.26 | 0.00 | 7.54E-05 |
| amt + amp + other.h + all.h + sage | 128.77 | 14.30 | 0.00 | 7.40E-05 |
| other.h + all.h + sage + soil | 128.91 | 14.44 | 0.00 | 6.90E-05 |
| amt + ants + ant.h + all.h + soil | 128.95 | 14.48 | 0.00 | 6.76E-05 |
| amt + amp + all.h + sage | 129.14 | 14.67 | 0.00 | 6.15E-05 |
| amt + amp + ant.h + other.h + soil | 129.17 | 14.70 | 0.00 | 6.04E-05 |
| amt + amp + ant.h + all.h + soil | 129.48 | 15.01 | 0.00 | 5.19E-05 |
| amt + amp + other.h + sage + soil | 129.48 | 15.01 | 0.00 | 5.18E-05 |
| amt + amp + ant.h + other.h + sage | 129.50 | 15.03 | 0.00 | 5.13E-05 |
| amt + amp + sage | 129.61 | 15.14 | 0.00 | 4.85E-05 |
| amt + amp + ant.h | 129.83 | 15.36 | 0.00 | 4.36E-05 |
| amt + sage + soil | 129.93 | 15.46 | 0.00 | 4.14E-05 |
| amt + amp + ant.h + all.h + sage | 130.12 | 15.65 | 0.00 | 3.76E-05 |
| amt + ant.h + other.h + all.h + sage + soil | 130.25 | 15.78 | 0.00 | 3.52E-05 |
| ant.h + all.h + sage | 130.26 | 15.79 | 0.00 | 3.50E-05 |
| amt + all.h + sage | 130.26 | 15.79 | 0.00 | 3.50E-05 |
| amt + amp + ant.h + other.h + all.h + soil | 130.27 | 15.80 | 0.00 | 3.49E-05 |
| amt + amp + all.h + sage + soil | 130.39 | 15.92 | 0.00 | 3.28E-05 |
| amt + amp + ant.h + soil | 130.40 | 15.93 | 0.00 | 3.26E-05 |
| amt + amp + other.h + all.h + sage + soil | 130.45 | 15.98 | 0.00 | 3.19E-05 |
| amt + amp + sage + soil | 130.54 | 16.07 | 0.00 | 3.05E-05 |
| ant.h + sage | 131.13 | 16.66 | 0.00 | 2.27E-05 |
| amt + amp + ant.h + other.h + sage + soil | 131.15 | 16.68 | 0.00 | 2.24E-05 |
| amt + amp + ant.h + sage | 131.33 | 16.86 | 0.00 | 2.05E-05 |
| amt + amp + ant.h + all.h + sage + soil | 131.47 | 17.00 | 0.00 | 1.91E-05 |
| amt + ant.h + sage + soil | 131.93 | 17.46 | 0.00 | 1.52E-05 |
| sage | 132.17 | 17.70 | 0.00 | 1.35E-05 |
| ant.h + all.h + sage + soil | 132.26 | 17.79 | 0.00 | 1.29E-05 |
| amt + amp + ant.h + other.h + all.h + sage + soil | 132.26 | 17.79 | 0.00 | 1.29E-05 |
| amt + amp + ant.h + sage + soil | 132.34 | 17.87 | 0.00 | 1.24E-05 |
| amt + other.h + sage | 133.08 | 18.61 | 0.00 | 8.57E-06 |
| ant.h + sage + soil | 133.13 | 18.66 | 0.00 | 8.34E-06 |
| sage + soil | 133.67 | 19.20 | 0.00 | 6.38E-06 |
| other.h | 133.77 | 19.30 | 0.00 | 6.05E-06 |
| amt + other.h | 134.24 | 19.77 | 0.00 | 4.79E-06 |
| amt + ant.h + other.h + sage | 134.31 | 19.84 | 0.00 | 4.63E-06 |
| other.h + sage | 134.44 | 19.97 | 0.00 | 4.33E-06 |
| amt + ant.h + other.h | 134.82 | 20.35 | 0.00 | 3.58E-06 |
| ant.h + other.h | 134.85 | 20.38 | 0.00 | 3.53E-06 |
| all.h + sage | 135.10 | 20.63 | 0.00 | 3.12E-06 |
| all.h | 135.10 | 20.63 | 0.00 | 3.11E-06 |
| all.h | 135.10 | 20.63 | 0.00 | 3.11E-06 |
| other.h + soil | 135.26 | 20.79 | 0.00 | 2.88E-06 |
| other.h + all.h | 135.62 | 21.15 | 0.00 | 2.40E-06 |
| ant.h + other.h + sage | 135.65 | 21.18 | 0.00 | 2.37E-06 |
| amt + ant.h + all.h + sage | 135.75 | 21.28 | 0.00 | 2.25E-06 |
| other.h + sage + soil | 135.80 | 21.33 | 0.00 | 2.20E-06 |
| ant.h | 135.88 | 21.41 | 0.00 | 2.11E-06 |
| amt + other.h + all.h | 136.03 | 21.56 | 0.00 | 1.96E-06 |
| amt + other.h + soil | 136.05 | 21.58 | 0.00 | 1.94E-06 |
| soil | 136.26 | 21.79 | 0.00 | 1.75E-06 |
| ant.h + other.h + soil | 136.29 | 21.82 | 0.00 | 1.72E-06 |
| other.h + all.h + sage | 136.37 | 21.90 | 0.00 | 1.65E-06 |
| amt + all.h | 136.43 | 21.96 | 0.00 | 1.60E-06 |
| amt + ant.h + other.h + all.h | 136.54 | 22.07 | 0.00 | 1.52E-06 |
| ant.h + other.h + sage + soil | 136.55 | 22.08 | 0.00 | 1.51E-06 |
| amt + ant.h + other.h + soil | 136.58 | 22.11 | 0.00 | 1.49E-06 |
| ant.h + other.h + all.h | 136.71 | 22.24 | 0.00 | 1.39E-06 |
| all.h + soil | 136.95 | 22.48 | 0.00 | 1.24E-06 |
| other.h + all.h + soil | 136.99 | 22.52 | 0.00 | 1.21E-06 |
| ant.h + all.h | 137.10 | 22.63 | 0.00 | 1.15E-06 |
| ant.h + other.h + all.h + sage | 137.40 | 22.93 | 0.00 | 9.89E-07 |
| amt + ant.h | 137.52 | 23.05 | 0.00 | 9.31E-07 |
| amt + other.h + all.h + soil | 137.75 | 23.28 | 0.00 | 8.29E-07 |
| ant.h + soil | 137.87 | 23.40 | 0.00 | 7.79E-07 |
| amt + soil | 137.97 | 23.50 | 0.00 | 7.42E-07 |
| ant.h + other.h + all.h + soil | 138.24 | 23.77 | 0.00 | 6.48E-07 |
| amt + all.h + soil | 138.39 | 23.92 | 0.00 | 6.01E-07 |
| amt + ant.h + other.h + all.h + soil | 138.40 | 23.93 | 0.00 | 6.00E-07 |
| amt + ant.h + all.h | 138.43 | 23.96 | 0.00 | 5.90E-07 |
| ant.h + other.h + all.h + sage + soil | 138.43 | 23.96 | 0.00 | 5.90E-07 |
| ant.h + all.h + soil | 138.94 | 24.47 | 0.00 | 4.57E-07 |
| amt + ant.h + soil | 139.51 | 25.04 | 0.00 | 3.44E-07 |
| amt + ant.h + all.h + soil | 140.39 | 25.92 | 0.00 | 2.21E-07 |

***^a^*** The environmental variables included in these models are annual mean temperature (amt), annual mean precipitation (amp), ant mound density per hectare (ants), diversity of non-ant arthropods in pitfall traps (other.h), diversity of ants in pitfall traps (ant.h), percentage of ground area that was sage (sage), survey start time (s.time), and temperature at the start of each survey (s.temp).

**Table S4.** Summary of results for abundance models. Abundance models (zero-eliminated) included the following variables: annual mean temperature (amt), annual mean precipitation (amp), elevation (elev), ant mound density per hectare (ants), the Shannon diversity of available ants (antH), the Shannon diversity of available non-ant invertebrates (otherH), the Shannon diversity of all available invertebrates (allH), the percentage of ground covered by sage (sage), start temperature of survey (s.temp), and start time of survey (s.time).

| Model structure^a^ | AICc | ΔAICc | AICω | R^2^ |
| --- | --- | --- | --- | --- |
| amt + amp + ants + s.temp*s.time | 156.47 | 0 | 1.26E-01 | 0.22 |
| amt*amp + ants + s.temp*s.time | 157.07 | 0.60 | 9.28E-02 | 0.23 |
| amt + amp + ants + sage + s.temp*s.time | 158.72 | 2.26 | 4.06E-02 | 0.21 |
| amt + amp + ants + soil + s.temp*s.time | 158.89 | 2.42 | 3.73E-02 | 0.21 |
| amt + amp + ants + all.h + s.temp*s.time | 159.08 | 2.61 | 3.41E-02 | 0.20 |
| amt + amp + ants + ant.h + s.temp*s.time | 159.22 | 2.75 | 3.17E-02 | 0.20 |
| amt + amp + ants + other.h + s.temp*s.time | 159.23 | 2.76 | 3.15E-02 | 0.20 |
| amt*amp + ants + sage + s.temp*s.time | 159.77 | 3.31 | 2.40E-02 | 0.22 |
| amt*amp + ants + all.h + s.temp*s.time | 159.79 | 3.32 | 2.39E-02 | 0.22 |
| amt*amp + ants + soil + s.temp*s.time | 159.87 | 3.40 | 2.29E-02 | 0.22 |
| amt*amp + ants + other.h + s.temp*s.time | 159.95 | 3.48 | 2.20E-02 | 0.21 |
| amt*amp + ants + ant.h + s.temp*s.time | 159.96 | 3.49 | 2.19E-02 | 0.21 |
| ants + s.temp*s.time | 160.10 | 3.63 | 2.04E-02 | 0.12 |
| amt*amp + ants * ant.h + s.temp*s.time | 160.55 | 4.08 | 1.63E-02 | 0.23 |
| amt + amp + ants * ant.h + s.temp*s.time | 160.93 | 4.46 | 1.35E-02 | 0.20 |
| amt + ants + s.temp*s.time | 161.26 | 4.80 | 1.14E-02 | 0.13 |
| amt + amp + ants + all.h + sage + s.temp*s.time | 161.31 | 4.84 | 1.11E-02 | 0.20 |
| amt + amp + ants + sage + soil + s.temp*s.time | 161.53 | 5.06 | 1.00E-02 | 0.19 |
| amt + amp + ants + all.h + soil + s.temp*s.time | 161.53 | 5.06 | 9.99E-03 | 0.19 |
| amt + amp + ants + other.h + sage + s.temp*s.time | 161.60 | 5.13 | 9.64E-03 | 0.19 |
| amt + amp + ants + ant.h + sage + s.temp*s.time | 161.60 | 5.13 | 9.63E-03 | 0.19 |
| amt + amp + ants + ant.h + soil + s.temp*s.time | 161.75 | 5.28 | 8.94E-03 | 0.19 |
| amt + amp + ants + other.h + soil + s.temp*s.time | 161.78 | 5.31 | 8.80E-03 | 0.19 |
| ants + all.h + s.temp*s.time | 161.87 | 5.40 | 8.42E-03 | 0.12 |
| amt + amp + ants + other.h + all.h + s.temp*s.time | 161.88 | 5.41 | 8.38E-03 | 0.19 |
| amt + amp + ants + ant.h + all.h + s.temp*s.time | 161.96 | 5.49 | 8.06E-03 | 0.19 |
| ants + sage + s.temp*s.time | 162.07 | 5.60 | 7.63E-03 | 0.12 |
| ants + soil + s.temp*s.time | 162.10 | 5.63 | 7.52E-03 | 0.12 |
| amt + amp + ants + ant.h + other.h + s.temp*s.time | 162.10 | 5.64 | 7.50E-03 | 0.19 |
| amt + ants + sage + s.temp*s.time | 162.13 | 5.66 | 7.40E-03 | 0.14 |
| amt*amp + ants + all.h + sage + s.temp*s.time | 162.53 | 6.07 | 6.05E-03 | 0.20 |
| ants + other.h + s.temp*s.time | 162.62 | 6.16 | 5.78E-03 | 0.11 |
| ants + ant.h + s.temp*s.time | 162.62 | 6.16 | 5.78E-03 | 0.11 |
| amt*amp + ants + all.h + soil + s.temp*s.time | 162.66 | 6.19 | 5.68E-03 | 0.20 |
| amt*amp + ants + ant.h + all.h + s.temp*s.time | 162.69 | 6.22 | 5.59E-03 | 0.20 |
| amt*amp + ants + other.h + sage + s.temp*s.time | 162.73 | 6.27 | 5.47E-03 | 0.20 |
| amt*amp + ants + sage + soil + s.temp*s.time | 162.77 | 6.30 | 5.37E-03 | 0.20 |
| amt*amp + ants + ant.h + sage + s.temp*s.time | 162.78 | 6.32 | 5.34E-03 | 0.20 |
| amt*amp + ants + other.h + all.h + s.temp*s.time | 162.79 | 6.32 | 5.31E-03 | 0.20 |
| amt*amp + ants * ant.h + all.h + s.temp*s.time | 162.81 | 6.34 | 5.27E-03 | 0.23 |
| amt*amp + ants + other.h + soil + s.temp*s.time | 162.85 | 6.39 | 5.15E-03 | 0.20 |
| amt*amp + ants + ant.h + soil + s.temp*s.time | 162.88 | 6.41 | 5.09E-03 | 0.20 |
| amt*amp + ants + ant.h + other.h + s.temp*s.time | 162.96 | 6.49 | 4.88E-03 | 0.20 |
| ants * ant.h + s.temp*s.time | 163.04 | 6.57 | 4.69E-03 | 0.12 |
| amt*amp + s.temp*s.time | 163.24 | 6.78 | 4.24E-03 | 0.12 |
| amt + amp + ants * ant.h + sage + s.temp*s.time | 163.29 | 6.82 | 4.14E-03 | 0.19 |
| amt*amp + ants * ant.h + sage + s.temp*s.time | 163.46 | 6.99 | 3.80E-03 | 0.22 |
| ants + all.h + sage + s.temp*s.time | 163.49 | 7.02 | 3.75E-03 | 0.12 |
| amt + ants + soil + s.temp*s.time | 163.53 | 7.06 | 3.68E-03 | 0.12 |
| amt + amp + ants * ant.h + all.h + s.temp*s.time | 163.54 | 7.07 | 3.65E-03 | 0.19 |
| amt + ants + all.h + s.temp*s.time | 163.58 | 7.11 | 3.58E-03 | 0.12 |
| amt*amp + ants * ant.h + other.h + s.temp*s.time | 163.60 | 7.13 | 3.55E-03 | 0.22 |
| ants + sage + soil + s.temp*s.time | 163.61 | 7.14 | 3.53E-03 | 0.12 |
| amt*amp + ants * ant.h + soil + s.temp*s.time | 163.68 | 7.22 | 3.40E-03 | 0.21 |
| amt + ants + sage + soil + s.temp*s.time | 163.69 | 7.22 | 3.39E-03 | 0.14 |
| all.h + s.temp*s.time | 163.69 | 7.23 | 3.39E-03 | 0.07 |
| all.h + s.temp*s.time | 163.69 | 7.23 | 3.39E-03 | 0.07 |
| amt + amp + ants * ant.h + soil + s.temp*s.time | 163.75 | 7.28 | 3.29E-03 | 0.19 |
| amt + ants + other.h + s.temp*s.time | 163.93 | 7.46 | 3.01E-03 | 0.11 |
| amt + ants + ant.h + s.temp*s.time | 163.93 | 7.47 | 3.00E-03 | 0.11 |
| amt + amp + ants * ant.h + other.h + s.temp*s.time | 163.94 | 7.47 | 2.99E-03 | 0.19 |
| ants + all.h + soil + s.temp*s.time | 164.18 | 7.71 | 2.66E-03 | 0.11 |
| amt + amp + ants + all.h + sage + soil + s.temp*s.time | 164.21 | 7.74 | 2.61E-03 | 0.18 |
| amt + amp + s.temp*s.time | 164.23 | 7.76 | 2.59E-03 | 0.08 |
| amt + amp + s.temp*s.time | 164.23 | 7.76 | 2.59E-03 | 0.08 |
| amt + ants + all.h + sage + s.temp*s.time | 164.24 | 7.77 | 2.58E-03 | 0.13 |
| amt + amp + ants + ant.h + all.h + sage + s.temp*s.time | 164.25 | 7.78 | 2.56E-03 | 0.18 |
| ants * ant.h + all.h + s.temp*s.time | 164.28 | 7.82 | 2.52E-03 | 0.13 |
| sage + s.temp*s.time | 164.28 | 7.82 | 2.52E-03 | 0.06 |
| amt + amp + ants + other.h + all.h + sage + s.temp*s.time | 164.31 | 7.85 | 2.48E-03 | 0.18 |
| amt + ants * ant.h + s.temp*s.time | 164.35 | 7.89 | 2.43E-03 | 0.13 |
| ants + ant.h + all.h + s.temp*s.time | 164.40 | 7.94 | 2.37E-03 | 0.10 |
| ants + other.h + sage + s.temp*s.time | 164.45 | 7.99 | 2.31E-03 | 0.10 |
| other.h + s.temp*s.time | 164.48 | 8.01 | 2.28E-03 | 0.06 |
| amt + amp + ants + other.h + all.h + soil + s.temp*s.time | 164.51 | 8.04 | 2.25E-03 | 0.18 |
| ants + other.h + all.h + s.temp*s.time | 164.51 | 8.05 | 2.25E-03 | 0.10 |
| soil + s.temp*s.time | 164.51 | 8.05 | 2.24E-03 | 0.06 |
| amt + amp + ants + other.h + sage + soil + s.temp*s.time | 164.52 | 8.05 | 2.24E-03 | 0.18 |
| ant.h + s.temp*s.time | 164.52 | 8.05 | 2.24E-03 | 0.06 |
| amt + amp + ants + ant.h + sage + soil + s.temp*s.time | 164.52 | 8.05 | 2.24E-03 | 0.18 |
| amt + amp + ants + ant.h + all.h + soil + s.temp*s.time | 164.52 | 8.06 | 2.24E-03 | 0.18 |
| amt + amp + ants + ant.h + other.h + sage + s.temp*s.time | 164.59 | 8.13 | 2.16E-03 | 0.18 |
| ants + ant.h + sage + s.temp*s.time | 164.68 | 8.21 | 2.07E-03 | 0.10 |
| ants + ant.h + soil + s.temp*s.time | 164.73 | 8.26 | 2.02E-03 | 0.10 |
| amt + amp + ants + ant.h + other.h + soil + s.temp*s.time | 164.76 | 8.29 | 1.99E-03 | 0.17 |
| ants + other.h + soil + s.temp*s.time | 164.76 | 8.30 | 1.98E-03 | 0.10 |
| amt + amp + ants + ant.h + other.h + all.h + s.temp*s.time | 164.79 | 8.32 | 1.96E-03 | 0.17 |
| amt + ants + other.h + sage + s.temp*s.time | 164.79 | 8.33 | 1.95E-03 | 0.12 |
| amt + ants + ant.h + sage + s.temp*s.time | 164.89 | 8.42 | 1.86E-03 | 0.12 |
| ants * ant.h + soil + s.temp*s.time | 165.09 | 8.62 | 1.68E-03 | 0.12 |
| ants * ant.h + sage + s.temp*s.time | 165.14 | 8.68 | 1.64E-03 | 0.12 |
| ants + ant.h + other.h + s.temp*s.time | 165.23 | 8.76 | 1.57E-03 | 0.09 |
| amt + ants * ant.h + sage + s.temp*s.time | 165.26 | 8.80 | 1.54E-03 | 0.14 |
| ants + all.h + sage + soil + s.temp*s.time | 165.38 | 8.92 | 1.45E-03 | 0.11 |
| amt*amp + all.h + s.temp*s.time | 165.40 | 8.93 | 1.44E-03 | 0.11 |
| all.h + sage + s.temp*s.time | 165.48 | 9.01 | 1.39E-03 | 0.06 |
| amt*amp + ants + ant.h + all.h + sage + s.temp*s.time | 165.49 | 9.02 | 1.38E-03 | 0.19 |
| amt*amp + ants * ant.h + all.h + sage + s.temp*s.time | 165.51 | 9.04 | 1.36E-03 | 0.22 |
| amt + amp + ants * ant.h + all.h + sage + s.temp*s.time | 165.61 | 9.15 | 1.30E-03 | 0.19 |
| amt*amp + ants + all.h + sage + soil + s.temp*s.time | 165.64 | 9.18 | 1.28E-03 | 0.19 |
| ants * ant.h + other.h + s.temp*s.time | 165.65 | 9.19 | 1.27E-03 | 0.11 |
| amt*amp + other.h + s.temp*s.time | 165.66 | 9.19 | 1.27E-03 | 0.11 |
| amt*amp + ants + other.h + all.h + sage + s.temp*s.time | 165.67 | 9.20 | 1.26E-03 | 0.19 |
| amt*amp + ants + ant.h + all.h + soil + s.temp*s.time | 165.69 | 9.22 | 1.25E-03 | 0.19 |
| ants * ant.h + all.h + sage + s.temp*s.time | 165.69 | 9.22 | 1.25E-03 | 0.14 |
| amt*amp + ants + ant.h + other.h + all.h + s.temp*s.time | 165.74 | 9.27 | 1.22E-03 | 0.19 |
| amt*amp + ants + other.h + all.h + soil + s.temp*s.time | 165.80 | 9.33 | 1.18E-03 | 0.19 |
| amt*amp + ants + other.h + sage + soil + s.temp*s.time | 165.85 | 9.38 | 1.15E-03 | 0.19 |
| amt*amp + ants + ant.h + other.h + sage + s.temp*s.time | 165.87 | 9.41 | 1.14E-03 | 0.19 |
| amt*amp + ants + ant.h + sage + soil + s.temp*s.time | 165.91 | 9.44 | 1.12E-03 | 0.18 |
| amt*amp + ants * ant.h + other.h + all.h + s.temp*s.time | 165.96 | 9.50 | 1.09E-03 | 0.21 |
| amt*amp + ant.h + s.temp*s.time | 165.99 | 9.52 | 1.08E-03 | 0.11 |
| amt*amp + ants + ant.h + other.h + soil + s.temp*s.time | 165.99 | 9.52 | 1.07E-03 | 0.18 |
| amt + sage + s.temp*s.time | 166.00 | 9.54 | 1.07E-03 | 0.06 |
| amt*amp + soil + s.temp*s.time | 166.01 | 9.54 | 1.06E-03 | 0.11 |
| ants + ant.h + all.h + sage + s.temp*s.time | 166.01 | 9.54 | 1.06E-03 | 0.11 |
| amt*amp + sage + s.temp*s.time | 166.01 | 9.54 | 1.06E-03 | 0.11 |
| amt + ants + all.h + soil + s.temp*s.time | 166.06 | 9.59 | 1.04E-03 | 0.10 |
| amt*amp + ants * ant.h + all.h + soil + s.temp*s.time | 166.08 | 9.62 | 1.02E-03 | 0.21 |
| amt + all.h + s.temp*s.time | 166.10 | 9.63 | 1.02E-03 | 0.06 |
| amt + ants + all.h + sage + soil + s.temp*s.time | 166.11 | 9.64 | 1.01E-03 | 0.13 |
| amt + ants + ant.h + all.h + s.temp*s.time | 166.15 | 9.68 | 9.91E-04 | 0.10 |
| amt + ants * ant.h + all.h + s.temp*s.time | 166.15 | 9.69 | 9.89E-04 | 0.13 |
| ant.h + all.h + s.temp*s.time | 166.20 | 9.74 | 9.64E-04 | 0.05 |
| all.h + soil + s.temp*s.time | 166.22 | 9.75 | 9.58E-04 | 0.05 |
| ants + other.h + sage + soil + s.temp*s.time | 166.22 | 9.76 | 9.55E-04 | 0.10 |
| amt + amp + all.h + s.temp*s.time | 166.23 | 9.77 | 9.51E-04 | 0.08 |
| ants + other.h + all.h + sage + s.temp*s.time | 166.25 | 9.78 | 9.44E-04 | 0.10 |
| amt + ants + other.h + all.h + s.temp*s.time | 166.25 | 9.78 | 9.42E-04 | 0.10 |
| amt + ants + other.h + soil + s.temp*s.time | 166.26 | 9.79 | 9.39E-04 | 0.10 |
| other.h + all.h + s.temp*s.time | 166.26 | 9.79 | 9.37E-04 | 0.05 |
| amt + ants * ant.h + all.h + sage + s.temp*s.time | 166.28 | 9.81 | 9.30E-04 | 0.15 |
| amt + ants + ant.h + soil + s.temp*s.time | 166.30 | 9.83 | 9.18E-04 | 0.10 |
| ants + ant.h + sage + soil + s.temp*s.time | 166.33 | 9.87 | 9.04E-04 | 0.10 |
| amt + amp + ants * ant.h + other.h + sage + s.temp*s.time | 166.35 | 9.88 | 8.97E-04 | 0.18 |
| other.h + sage + s.temp*s.time | 166.37 | 9.90 | 8.89E-04 | 0.05 |
| amt + amp + ants * ant.h + all.h + soil + s.temp*s.time | 166.41 | 9.94 | 8.71E-04 | 0.18 |
| amt + amp + ants * ant.h + sage + soil + s.temp*s.time | 166.42 | 9.96 | 8.63E-04 | 0.18 |
| amt + amp + ants * ant.h + other.h + all.h + s.temp*s.time | 166.45 | 9.99 | 8.52E-04 | 0.18 |
| sage + soil + s.temp*s.time | 166.50 | 10.03 | 8.32E-04 | 0.05 |
| amt*amp + ants * ant.h + other.h + sage + s.temp*s.time | 166.52 | 10.06 | 8.21E-04 | 0.20 |
| amt + ants + other.h + sage + soil + s.temp*s.time | 166.54 | 10.08 | 8.14E-04 | 0.12 |
| amt + ants + ant.h + sage + soil + s.temp*s.time | 166.56 | 10.09 | 8.07E-04 | 0.12 |
| amt + ants + ant.h + all.h + sage + s.temp*s.time | 166.62 | 10.16 | 7.82E-04 | 0.12 |
| amt*amp + ants * ant.h + sage + soil + s.temp*s.time | 166.64 | 10.17 | 7.75E-04 | 0.20 |
| ants * ant.h + sage + soil + s.temp*s.time | 166.64 | 10.18 | 7.75E-04 | 0.12 |
| amt + amp + soil + s.temp*s.time | 166.67 | 10.20 | 7.63E-04 | 0.07 |
| amt + amp + sage + s.temp*s.time | 166.68 | 10.21 | 7.60E-04 | 0.07 |
| ant.h + sage + s.temp*s.time | 166.69 | 10.22 | 7.57E-04 | 0.05 |
| amt + ants * ant.h + soil + s.temp*s.time | 166.70 | 10.23 | 7.53E-04 | 0.12 |
| amt + amp + ant.h + s.temp*s.time | 166.70 | 10.23 | 7.53E-04 | 0.07 |
| amt + ants + ant.h + other.h + s.temp*s.time | 166.70 | 10.23 | 7.53E-04 | 0.09 |
| amt + soil + s.temp*s.time | 166.73 | 10.27 | 7.40E-04 | 0.05 |
| amt + other.h + s.temp*s.time | 166.73 | 10.27 | 7.40E-04 | 0.05 |
| amt + ants * ant.h + sage + soil + s.temp*s.time | 166.75 | 10.28 | 7.35E-04 | 0.15 |
| amt + ant.h + s.temp*s.time | 166.76 | 10.29 | 7.31E-04 | 0.04 |
| amt + amp + other.h + s.temp*s.time | 166.76 | 10.30 | 7.30E-04 | 0.07 |
| ants * ant.h + all.h + soil + s.temp*s.time | 166.79 | 10.32 | 7.19E-04 | 0.12 |
| ant.h + other.h + s.temp*s.time | 166.85 | 10.38 | 6.98E-04 | 0.04 |
| ants + ant.h + all.h + soil + s.temp*s.time | 166.86 | 10.39 | 6.95E-04 | 0.09 |
| amt + amp + ants * ant.h + other.h + soil + s.temp*s.time | 166.87 | 10.40 | 6.91E-04 | 0.17 |
| amt*amp + ants * ant.h + other.h + soil + s.temp*s.time | 166.87 | 10.41 | 6.90E-04 | 0.20 |
| ants + other.h + all.h + soil + s.temp*s.time | 166.88 | 10.41 | 6.88E-04 | 0.09 |
| ants * ant.h + other.h + all.h + s.temp*s.time | 166.94 | 10.47 | 6.67E-04 | 0.12 |
| ant.h + soil + s.temp*s.time | 166.95 | 10.48 | 6.65E-04 | 0.04 |
| other.h + soil + s.temp*s.time | 166.96 | 10.49 | 6.61E-04 | 0.04 |
| ants + ant.h + other.h + all.h + s.temp*s.time | 166.98 | 10.52 | 6.53E-04 | 0.09 |
| ants + ant.h + other.h + sage + s.temp*s.time | 167.10 | 10.64 | 6.15E-04 | 0.09 |
| amt + ants + other.h + all.h + sage + s.temp*s.time | 167.12 | 10.66 | 6.09E-04 | 0.11 |
| amt + ants * ant.h + other.h + s.temp*s.time | 167.24 | 10.77 | 5.75E-04 | 0.11 |
| amt + amp + ants + ant.h + all.h + sage + soil + s.temp*s.time | 167.28 | 10.82 | 5.62E-04 | 0.17 |
| amt + amp + ants + ant.h + other.h + all.h + sage + s.temp*s.time | 167.31 | 10.85 | 5.54E-04 | 0.17 |
| amt + amp + ants + other.h + all.h + sage + soil + s.temp*s.time | 167.34 | 10.88 | 5.45E-04 | 0.16 |
| ants * ant.h + other.h + sage + s.temp*s.time | 167.44 | 10.97 | 5.20E-04 | 0.11 |
| ants + ant.h + other.h + soil + s.temp*s.time | 167.50 | 11.03 | 5.05E-04 | 0.08 |
| amt + amp + ants + ant.h + other.h + all.h + soil + s.temp*s.time | 167.57 | 11.10 | 4.87E-04 | 0.16 |
| amt + all.h + sage + s.temp*s.time | 167.63 | 11.16 | 4.73E-04 | 0.06 |
| amt + amp + ants + ant.h + other.h + sage + soil + s.temp*s.time | 167.63 | 11.16 | 4.73E-04 | 0.16 |
| amt + ants + ant.h + other.h + sage + s.temp*s.time | 167.68 | 11.21 | 4.61E-04 | 0.11 |
| ants * ant.h + all.h + sage + soil + s.temp*s.time | 167.69 | 11.23 | 4.58E-04 | 0.13 |
| all.h + sage + soil + s.temp*s.time | 167.93 | 11.46 | 4.08E-04 | 0.05 |
| ants * ant.h + other.h + soil + s.temp*s.time | 167.93 | 11.47 | 4.06E-04 | 0.10 |
| ant.h + all.h + sage + s.temp*s.time | 168.02 | 11.56 | 3.88E-04 | 0.05 |
| amt + ants * ant.h + other.h + sage + s.temp*s.time | 168.03 | 11.56 | 3.87E-04 | 0.13 |
| other.h + all.h + sage + s.temp*s.time | 168.06 | 11.60 | 3.81E-04 | 0.05 |
| ants + ant.h + all.h + sage + soil + s.temp*s.time | 168.08 | 11.61 | 3.78E-04 | 0.10 |
| amt*amp + ant.h + all.h + s.temp*s.time | 168.18 | 11.72 | 3.59E-04 | 0.10 |
| amt*amp + other.h + all.h + s.temp*s.time | 168.18 | 11.72 | 3.58E-04 | 0.10 |
| amt*amp + all.h + sage + s.temp*s.time | 168.22 | 11.75 | 3.52E-04 | 0.10 |
| amt*amp + all.h + soil + s.temp*s.time | 168.23 | 11.76 | 3.50E-04 | 0.10 |
| amt + ants * ant.h + all.h + sage + soil + s.temp*s.time | 168.27 | 11.80 | 3.43E-04 | 0.15 |
| ants + other.h + all.h + sage + soil + s.temp*s.time | 168.27 | 11.80 | 3.43E-04 | 0.10 |
| amt + other.h + sage + s.temp*s.time | 168.30 | 11.83 | 3.38E-04 | 0.04 |
| amt + sage + soil + s.temp*s.time | 168.36 | 11.89 | 3.29E-04 | 0.04 |
| amt*amp + ant.h + other.h + s.temp*s.time | 168.45 | 11.99 | 3.13E-04 | 0.09 |
| amt*amp + other.h + sage + s.temp*s.time | 168.46 | 12.00 | 3.12E-04 | 0.09 |
| amt*amp + other.h + soil + s.temp*s.time | 168.49 | 12.03 | 3.07E-04 | 0.09 |
| amt + ants + ant.h + other.h + all.h + s.temp*s.time | 168.54 | 12.07 | 3.00E-04 | 0.09 |
| amt + amp + all.h + sage + s.temp*s.time | 168.61 | 12.14 | 2.90E-04 | 0.07 |
| amt + ants * ant.h + other.h + all.h + s.temp*s.time | 168.61 | 12.14 | 2.90E-04 | 0.12 |
| amt + ant.h + sage + s.temp*s.time | 168.62 | 12.15 | 2.88E-04 | 0.04 |
| amt + amp + all.h + soil + s.temp*s.time | 168.64 | 12.17 | 2.85E-04 | 0.07 |
| ants * ant.h + other.h + all.h + sage + s.temp*s.time | 168.67 | 12.21 | 2.80E-04 | 0.12 |
| amt + ants + ant.h + all.h + sage + soil + s.temp*s.time | 168.69 | 12.23 | 2.77E-04 | 0.12 |
| amt + ant.h + all.h + s.temp*s.time | 168.70 | 12.23 | 2.77E-04 | 0.04 |
| amt*amp + ants + ant.h + other.h + all.h + sage + s.temp*s.time | 168.72 | 12.25 | 2.74E-04 | 0.17 |
| amt + all.h + soil + s.temp*s.time | 168.74 | 12.27 | 2.72E-04 | 0.04 |
| amt*amp + ants + ant.h + all.h + sage + soil + s.temp*s.time | 168.74 | 12.28 | 2.71E-04 | 0.17 |
| ant.h + other.h + sage + s.temp*s.time | 168.74 | 12.28 | 2.71E-04 | 0.04 |
| other.h + sage + soil + s.temp*s.time | 168.76 | 12.30 | 2.68E-04 | 0.04 |
| amt + other.h + all.h + s.temp*s.time | 168.77 | 12.30 | 2.68E-04 | 0.04 |
| amt + ants + other.h + all.h + soil + s.temp*s.time | 168.77 | 12.30 | 2.67E-04 | 0.09 |
| amt + ants + ant.h + all.h + soil + s.temp*s.time | 168.79 | 12.32 | 2.65E-04 | 0.09 |
| amt + amp + ants * ant.h + other.h + all.h + sage + s.temp*s.time | 168.80 | 12.33 | 2.64E-04 | 0.17 |
| amt*amp + ants * ant.h + all.h + sage + soil + s.temp*s.time | 168.80 | 12.34 | 2.63E-04 | 0.20 |
| ant.h + all.h + soil + s.temp*s.time | 168.84 | 12.37 | 2.58E-04 | 0.04 |
| ant.h + other.h + all.h + s.temp*s.time | 168.86 | 12.39 | 2.56E-04 | 0.04 |
| amt + ants * ant.h + all.h + soil + s.temp*s.time | 168.86 | 12.39 | 2.56E-04 | 0.12 |
| amt*amp + ant.h + soil + s.temp*s.time | 168.86 | 12.39 | 2.55E-04 | 0.09 |
| amt*amp + ant.h + sage + s.temp*s.time | 168.87 | 12.40 | 2.55E-04 | 0.09 |
| ants + ant.h + other.h + all.h + sage + s.temp*s.time | 168.87 | 12.41 | 2.54E-04 | 0.09 |
| amt*amp + ants * ant.h + other.h + all.h + sage + s.temp*s.time | 168.88 | 12.41 | 2.53E-04 | 0.20 |
| amt + amp + ants * ant.h + all.h + sage + soil + s.temp*s.time | 168.88 | 12.42 | 2.52E-04 | 0.17 |
| other.h + all.h + soil + s.temp*s.time | 168.89 | 12.42 | 2.52E-04 | 0.04 |
| amt*amp + sage + soil + s.temp*s.time | 168.89 | 12.43 | 2.51E-04 | 0.09 |
| amt*amp + ants + ant.h + other.h + all.h + soil + s.temp*s.time | 168.90 | 12.43 | 2.50E-04 | 0.17 |
| amt*amp + ants + other.h + all.h + sage + soil + s.temp*s.time | 168.91 | 12.45 | 2.49E-04 | 0.17 |
| ant.h + sage + soil + s.temp*s.time | 169.00 | 12.54 | 2.38E-04 | 0.03 |
| amt + amp + other.h + all.h + s.temp*s.time | 169.00 | 12.54 | 2.38E-04 | 0.06 |
| amt + amp + ant.h + all.h + s.temp*s.time | 169.01 | 12.54 | 2.37E-04 | 0.06 |
| ants + ant.h + other.h + sage + soil + s.temp*s.time | 169.01 | 12.55 | 2.37E-04 | 0.09 |
| amt + ants + other.h + all.h + sage + soil + s.temp*s.time | 169.11 | 12.65 | 2.25E-04 | 0.11 |
| amt*amp + ants + ant.h + other.h + sage + soil + s.temp*s.time | 169.13 | 12.66 | 2.24E-04 | 0.17 |
| amt + ants + ant.h + other.h + soil + s.temp*s.time | 169.14 | 12.67 | 2.23E-04 | 0.08 |
| amt + amp + other.h + sage + s.temp*s.time | 169.16 | 12.69 | 2.20E-04 | 0.06 |
| amt + amp + other.h + soil + s.temp*s.time | 169.21 | 12.74 | 2.15E-04 | 0.06 |
| amt + amp + ant.h + soil + s.temp*s.time | 169.23 | 12.76 | 2.12E-04 | 0.06 |
| amt + ants * ant.h + other.h + all.h + sage + s.temp*s.time | 169.24 | 12.78 | 2.11E-04 | 0.14 |
| amt + amp + ant.h + other.h + s.temp*s.time | 169.25 | 12.78 | 2.10E-04 | 0.06 |
| ants * ant.h + other.h + sage + soil + s.temp*s.time | 169.25 | 12.79 | 2.10E-04 | 0.11 |
| amt + amp + ant.h + sage + s.temp*s.time | 169.28 | 12.81 | 2.07E-04 | 0.06 |
| amt + ant.h + other.h + s.temp*s.time | 169.30 | 12.84 | 2.05E-04 | 0.03 |
| amt + ant.h + soil + s.temp*s.time | 169.34 | 12.87 | 2.01E-04 | 0.03 |
| amt + other.h + soil + s.temp*s.time | 169.34 | 12.88 | 2.01E-04 | 0.03 |
| amt + amp + sage + soil + s.temp*s.time | 169.37 | 12.90 | 1.98E-04 | 0.05 |
| amt*amp + ants * ant.h + other.h + all.h + soil + s.temp*s.time | 169.38 | 12.92 | 1.97E-04 | 0.19 |
| ant.h + other.h + soil + s.temp*s.time | 169.44 | 12.98 | 1.91E-04 | 0.03 |
| amt + ants + ant.h + other.h + all.h + sage + s.temp*s.time | 169.47 | 13.01 | 1.88E-04 | 0.11 |
| ants + ant.h + other.h + all.h + soil + s.temp*s.time | 169.47 | 13.01 | 1.88E-04 | 0.08 |
| ants * ant.h + other.h + all.h + soil + s.temp*s.time | 169.48 | 13.01 | 1.88E-04 | 0.11 |
| amt + amp + ants * ant.h + other.h + all.h + soil + s.temp*s.time | 169.53 | 13.07 | 1.82E-04 | 0.16 |
| amt + ants + ant.h + other.h + sage + soil + s.temp*s.time | 169.54 | 13.08 | 1.82E-04 | 0.11 |
| amt + amp + ants * ant.h + other.h + sage + soil + s.temp*s.time | 169.62 | 13.15 | 1.75E-04 | 0.16 |
| amt + ants * ant.h + other.h + soil + s.temp*s.time | 169.70 | 13.23 | 1.68E-04 | 0.10 |
| amt + ants * ant.h + other.h + sage + soil + s.temp*s.time | 169.77 | 13.31 | 1.62E-04 | 0.13 |
| amt*amp + ants * ant.h + other.h + sage + soil + s.temp*s.time | 169.87 | 13.40 | 1.54E-04 | 0.19 |
| amt + all.h + sage + soil + s.temp*s.time | 170.19 | 13.73 | 1.31E-04 | 0.04 |
| amt + ant.h + all.h + sage + s.temp*s.time | 170.21 | 13.75 | 1.30E-04 | 0.04 |
| amt + other.h + all.h + sage + s.temp*s.time | 170.33 | 13.87 | 1.22E-04 | 0.04 |
| amt + amp + ants + ant.h + other.h + all.h + sage + soil + s.temp*s.time | 170.50 | 14.04 | 1.12E-04 | 0.15 |
| ant.h + all.h + sage + soil + s.temp*s.time | 170.60 | 14.14 | 1.07E-04 | 0.03 |
| other.h + all.h + sage + soil + s.temp*s.time | 170.64 | 14.17 | 1.05E-04 | 0.03 |
| ants * ant.h + other.h + all.h + sage + soil + s.temp*s.time | 170.78 | 14.31 | 9.78E-05 | 0.12 |
| ant.h + other.h + all.h + sage + s.temp*s.time | 170.78 | 14.32 | 9.76E-05 | 0.03 |
| amt + other.h + sage + soil + s.temp*s.time | 170.82 | 14.36 | 9.57E-05 | 0.03 |
| amt + ant.h + other.h + sage + s.temp*s.time | 170.94 | 14.48 | 9.01E-05 | 0.03 |
| ants + ant.h + other.h + all.h + sage + soil + s.temp*s.time | 171.04 | 14.58 | 8.58E-05 | 0.08 |
| amt*amp + other.h + all.h + sage + s.temp*s.time | 171.07 | 14.61 | 8.46E-05 | 0.08 |
| amt + ant.h + sage + soil + s.temp*s.time | 171.08 | 14.61 | 8.43E-05 | 0.03 |
| amt*amp + ant.h + all.h + sage + s.temp*s.time | 171.09 | 14.62 | 8.39E-05 | 0.08 |
| amt*amp + other.h + all.h + soil + s.temp*s.time | 171.11 | 14.65 | 8.29E-05 | 0.08 |
| amt*amp + ant.h + all.h + soil + s.temp*s.time | 171.13 | 14.67 | 8.19E-05 | 0.08 |
| amt*amp + ant.h + other.h + all.h + s.temp*s.time | 171.16 | 14.70 | 8.07E-05 | 0.08 |
| amt + ants + ant.h + other.h + all.h + soil + s.temp*s.time | 171.20 | 14.73 | 7.93E-05 | 0.08 |
| amt*amp + all.h + sage + soil + s.temp*s.time | 171.21 | 14.74 | 7.89E-05 | 0.08 |
| ant.h + other.h + sage + soil + s.temp*s.time | 171.26 | 14.79 | 7.71E-05 | 0.02 |
| amt + ants * ant.h + other.h + all.h + sage + soil + s.temp*s.time | 171.31 | 14.84 | 7.52E-05 | 0.14 |
| amt + ants * ant.h + other.h + all.h + soil + s.temp*s.time | 171.33 | 14.86 | 7.44E-05 | 0.11 |
| amt + amp + all.h + sage + soil + s.temp*s.time | 171.37 | 14.90 | 7.29E-05 | 0.05 |
| amt*amp + ant.h + other.h + sage + s.temp*s.time | 171.37 | 14.90 | 7.28E-05 | 0.08 |
| amt*amp + ant.h + other.h + soil + s.temp*s.time | 171.39 | 14.92 | 7.22E-05 | 0.08 |
| amt + ant.h + other.h + all.h + s.temp*s.time | 171.42 | 14.96 | 7.09E-05 | 0.02 |
| amt + amp + other.h + all.h + sage + s.temp*s.time | 171.44 | 14.98 | 7.03E-05 | 0.05 |
| amt + ant.h + all.h + soil + s.temp*s.time | 171.45 | 14.99 | 6.99E-05 | 0.02 |
| amt*amp + other.h + sage + soil + s.temp*s.time | 171.46 | 14.99 | 6.98E-05 | 0.08 |
| amt + amp + ant.h + all.h + sage + s.temp*s.time | 171.48 | 15.01 | 6.91E-05 | 0.05 |
| amt + amp + other.h + all.h + soil + s.temp*s.time | 171.50 | 15.03 | 6.83E-05 | 0.05 |
| amt + other.h + all.h + soil + s.temp*s.time | 171.51 | 15.04 | 6.79E-05 | 0.02 |
| amt + amp + ant.h + all.h + soil + s.temp*s.time | 171.53 | 15.06 | 6.73E-05 | 0.05 |
| ant.h + other.h + all.h + soil + s.temp*s.time | 171.59 | 15.12 | 6.52E-05 | 0.02 |
| amt + ants + ant.h + other.h + all.h + sage + soil + s.temp*s.time | 171.61 | 15.15 | 6.45E-05 | 0.10 |
| amt + amp + ant.h + other.h + soil + s.temp*s.time | 171.75 | 15.28 | 6.04E-05 | 0.04 |
| amt + amp + ant.h + other.h + sage + s.temp*s.time | 171.76 | 15.29 | 6.01E-05 | 0.04 |
| amt*amp + ant.h + sage + soil + s.temp*s.time | 171.87 | 15.40 | 5.68E-05 | 0.07 |
| amt + amp + ant.h + other.h + all.h + s.temp*s.time | 171.89 | 15.43 | 5.60E-05 | 0.04 |
| amt + amp + other.h + sage + soil + s.temp*s.time | 171.93 | 15.46 | 5.51E-05 | 0.04 |
| amt + ant.h + other.h + soil + s.temp*s.time | 172.02 | 15.55 | 5.26E-05 | 0.01 |
| amt + amp + ant.h + sage + soil + s.temp*s.time | 172.06 | 15.60 | 5.15E-05 | 0.04 |
| amt*amp + ants + ant.h + other.h + all.h + sage + soil + s.temp*s.time | 172.12 | 15.66 | 4.99E-05 | 0.16 |
| amt + amp + ants * ant.h + other.h + all.h + sage + soil + s.temp*s.time | 172.22 | 15.75 | 4.77E-05 | 0.16 |
| amt*amp + ants * ant.h + other.h + all.h + sage + soil + s.temp*s.time | 172.31 | 15.84 | 4.55E-05 | 0.19 |
| amt + ant.h + all.h + sage + soil + s.temp*s.time | 172.92 | 16.46 | 3.35E-05 | 0.02 |
| amt + other.h + all.h + sage + soil + s.temp*s.time | 173.03 | 16.56 | 3.18E-05 | 0.02 |
| amt + ant.h + other.h + all.h + sage + s.temp*s.time | 173.10 | 16.64 | 3.06E-05 | 0.02 |
| ant.h + other.h + all.h + sage + soil + s.temp*s.time | 173.48 | 17.02 | 2.53E-05 | 0.02 |
| amt + ant.h + other.h + sage + soil + s.temp*s.time | 173.59 | 17.12 | 2.40E-05 | 0.01 |
| amt*amp + ant.h + other.h + all.h + sage + s.temp*s.time | 174.17 | 17.71 | 1.79E-05 | 0.06 |
| amt*amp + other.h + all.h + sage + soil + s.temp*s.time | 174.18 | 17.72 | 1.78E-05 | 0.06 |
| amt*amp + ant.h + all.h + sage + soil + s.temp*s.time | 174.21 | 17.75 | 1.76E-05 | 0.06 |
| amt*amp + ant.h + other.h + all.h + soil + s.temp*s.time | 174.23 | 17.76 | 1.75E-05 | 0.00 |
| amt + ant.h + other.h + all.h + soil + s.temp*s.time | 174.29 | 17.82 | 1.69E-05 | 0.03 |
| amt + amp + other.h + all.h + sage + soil + s.temp*s.time | 174.31 | 17.84 | 1.68E-05 | 0.03 |
| amt + amp + ant.h + all.h + sage + soil + s.temp*s.time | 174.36 | 17.89 | 1.63E-05 | 0.03 |
| amt + amp + ant.h + other.h + all.h + sage + s.temp*s.time | 174.45 | 17.98 | 1.56E-05 | 0.06 |
| amt*amp + ant.h + other.h + sage + soil + s.temp*s.time | 174.48 | 18.02 | 1.54E-05 | 0.03 |
| amt + amp + ant.h + other.h + all.h + soil + s.temp*s.time | 174.51 | 18.04 | 1.52E-05 | 0.03 |
| amt + amp + ant.h + other.h + sage + soil + s.temp*s.time | 174.61 | 18.14 | 1.44E-05 | 0.00 |
| amt + ant.h + other.h + all.h + sage + soil + s.temp*s.time | 175.93 | 19.47 | 7.43E-06 | 0.04 |
| amt*amp + ant.h + other.h + all.h + sage + soil + s.temp*s.time | 177.43 | 20.96 | 3.52E-06 | 0.01 |
| amt + amp + ant.h + other.h + all.h + sage + soil + s.temp*s.time | 177.45 | 20.98 | 3.49E-06 | 0.01 |

***^a^*** The environmental variables included in these models are annual mean temperature (amt), annual mean precipitation (amp), ant mound density per hectare (ants), diversity of non-ant arthropods in pitfall traps (other.h), diversity of ants in pitfall traps (ant.h), percentage of ground area that was sage (sage), survey start time (s.time), and temperature at the start of each survey (s.temp).

**Table S5.** Summary of results for linear mixed models with Poisson link. Abundance models (including zero) included the following variables: annual mean temperature (amt), annual mean precipitation (amp), elevation (elev), ant mound density per hectare (ants), the Shannon diversity of available ants (antH), the Shannon diversity of available non-ant invertebrates (otherH), the Shannon diversity of all available invertebrates (allH), the percentage of ground covered by sage (sage), start temperature of survey (s.temp), and start time of survey (s.time).

| Model structure^a^ | AICc | ΔAIC | AICω |
| --- | --- | --- | --- |
| ants.r + all.h + s.temp*s.time | 399.84 | 0.00 | 8.01E-02 |
| ants.r + s.temp*s.time | 400.57 | 0.74 | 5.55E-02 |
| amt + ants.r + other.h + soil + s.temp*s.time | 401.26 | 1.43 | 3.93E-02 |
| ants.r + ant.h + all.h + s.temp*s.time | 401.44 | 1.61 | 3.59E-02 |
| amt + ant.h + soil + s.temp*s.time | 401.47 | 1.63 | 3.55E-02 |
| ants.r + all.h + sage + s.temp*s.time | 401.73 | 1.89 | 3.12E-02 |
| ants.r * ant.h + all.h + s.temp*s.time | 402.12 | 2.28 | 2.56E-02 |
| ants.r + other.h + all.h + s.temp*s.time | 402.13 | 2.29 | 2.55E-02 |
| ants.r + all.h + soil + s.temp*s.time | 402.21 | 2.37 | 2.45E-02 |
| ants.r + soil + s.temp*s.time | 402.74 | 2.91 | 1.87E-02 |
| ants.r + sage + s.temp*s.time | 402.75 | 2.92 | 1.86E-02 |
| ants.r + other.h + s.temp*s.time | 402.80 | 2.97 | 1.82E-02 |
| ants.r + ant.h + s.temp*s.time | 402.88 | 3.04 | 1.75E-02 |
| amt + amp + ants.r + sage + soil + s.temp*s.time | 403.03 | 3.20 | 1.62E-02 |
| amt + ant.h + sage + soil + s.temp*s.time | 403.06 | 3.22 | 1.60E-02 |
| amt + ants.r + other.h + sage + soil + s.temp*s.time | 403.10 | 3.26 | 1.57E-02 |
| amt + ants.r + all.h + soil + s.temp*s.time | 403.18 | 3.34 | 1.51E-02 |
| ants.r + ant.h + other.h + all.h + s.temp*s.time | 403.18 | 3.34 | 1.51E-02 |
| ants.r + ant.h + all.h + sage + s.temp*s.time | 403.30 | 3.46 | 1.42E-02 |
| amt + amp + ants.r + all.h + sage + s.temp*s.time | 403.35 | 3.51 | 1.38E-02 |
| amt + ants.r + other.h + sage + s.temp*s.time | 403.64 | 3.80 | 1.20E-02 |
| amt + ants.r + ant.h + other.h + soil + s.temp*s.time | 403.67 | 3.83 | 1.18E-02 |
| amt + ants.r + other.h + all.h + soil + s.temp*s.time | 403.67 | 3.83 | 1.18E-02 |
| amt + ants.r * ant.h + soil + s.temp*s.time | 403.69 | 3.85 | 1.17E-02 |
| ants.r + ant.h + all.h + soil + s.temp*s.time | 403.90 | 4.06 | 1.05E-02 |
| amt + ant.h + sage + s.temp*s.time | 403.93 | 4.10 | 1.03E-02 |
| ants.r * ant.h + all.h + sage + s.temp*s.time | 403.95 | 4.11 | 1.03E-02 |
| ants.r + all.h + sage + soil + s.temp*s.time | 403.96 | 4.12 | 1.02E-02 |
| ants.r * ant.h + other.h + all.h + s.temp*s.time | 404.00 | 4.16 | 1.00E-02 |
| amt*amp + ants.r + s.temp*s.time | 404.02 | 4.18 | 9.92E-03 |
| amt + ants.r + ant.h + soil + s.temp*s.time | 404.05 | 4.21 | 9.74E-03 |
| ants.r + other.h + all.h + sage + s.temp*s.time | 404.18 | 4.34 | 9.14E-03 |
| ants.r * ant.h + s.temp*s.time | 404.21 | 4.38 | 8.99E-03 |
| amt*amp + ants.r + all.h + s.temp*s.time | 404.28 | 4.44 | 8.70E-03 |
| ants.r + other.h + all.h + soil + s.temp*s.time | 404.54 | 4.70 | 7.65E-03 |
| ants.r * ant.h + all.h + soil + s.temp*s.time | 404.63 | 4.79 | 7.31E-03 |
| amt + ants.r + ant.h + all.h + soil + s.temp*s.time | 404.64 | 4.80 | 7.26E-03 |
| amt + ants.r + all.h + sage + soil + s.temp*s.time | 404.70 | 4.86 | 7.05E-03 |
| ants.r + sage + soil + s.temp*s.time | 404.78 | 4.94 | 6.78E-03 |
| ants.r + other.h + sage + s.temp*s.time | 404.89 | 5.05 | 6.41E-03 |
| ants.r + other.h + soil + s.temp*s.time | 405.08 | 5.24 | 5.83E-03 |
| ants.r + ant.h + sage + s.temp*s.time | 405.11 | 5.27 | 5.75E-03 |
| ants.r + ant.h + soil + s.temp*s.time | 405.11 | 5.27 | 5.75E-03 |
| ants.r + ant.h + other.h + s.temp*s.time | 405.14 | 5.30 | 5.65E-03 |
| amt + ants.r * ant.h + other.h + soil + s.temp*s.time | 405.15 | 5.31 | 5.63E-03 |
| amt + amp + ants.r + other.h + soil + s.temp*s.time | 405.18 | 5.34 | 5.55E-03 |
| amt + amp + ants.r + ant.h + all.h + sage + s.temp*s.time | 405.19 | 5.36 | 5.50E-03 |
| amt + amp + ant.h + s.temp*s.time | 405.28 | 5.44 | 5.28E-03 |
| amt + ants.r + other.h + all.h + s.temp*s.time | 405.36 | 5.52 | 5.07E-03 |
| ants.r + ant.h + other.h + all.h + sage + s.temp*s.time | 405.40 | 5.56 | 4.97E-03 |
| amt + ant.h + other.h + s.temp*s.time | 405.45 | 5.61 | 4.85E-03 |
| amt + amp + ants.r + ant.h + sage + s.temp*s.time | 405.49 | 5.65 | 4.76E-03 |
| amt + ants.r + other.h + all.h + sage + soil + s.temp*s.time | 405.49 | 5.65 | 4.76E-03 |
| amt + amp + ants.r + other.h + sage + soil + s.temp*s.time | 405.49 | 5.65 | 4.74E-03 |
| amt + amp + ants.r + other.h + s.temp*s.time | 405.50 | 5.66 | 4.72E-03 |
| amt + ants.r * ant.h + sage + soil + s.temp*s.time | 405.53 | 5.70 | 4.65E-03 |
| amt + ants.r + ant.h + sage + soil + s.temp*s.time | 405.56 | 5.72 | 4.58E-03 |
| amt + ants.r + ant.h + other.h + sage + soil + s.temp*s.time | 405.57 | 5.73 | 4.57E-03 |
| ants.r + ant.h + all.h + sage + soil + s.temp*s.time | 405.64 | 5.80 | 4.41E-03 |
| amt + amp + ants.r + other.h + all.h + sage + soil + s.temp*s.time | 405.66 | 5.82 | 4.36E-03 |
| amt + ants.r * ant.h + all.h + sage + s.temp*s.time | 405.69 | 5.85 | 4.30E-03 |
| ants.r + ant.h + other.h + all.h + soil + s.temp*s.time | 405.69 | 5.85 | 4.29E-03 |
| amt + ants.r + all.h + sage + s.temp*s.time | 405.71 | 5.87 | 4.25E-03 |
| amt + amp + ants.r + all.h + sage + soil + s.temp*s.time | 405.81 | 5.97 | 4.05E-03 |
| amt*amp + ants.r * ant.h + all.h + s.temp*s.time | 405.83 | 5.99 | 4.01E-03 |
| amt*amp + ants.r + ant.h + all.h + s.temp*s.time | 405.84 | 6.00 | 3.99E-03 |
| amt + ants.r + ant.h + other.h + sage + s.temp*s.time | 406.11 | 6.27 | 3.49E-03 |
| amt + ants.r + other.h + all.h + sage + s.temp*s.time | 406.11 | 6.28 | 3.48E-03 |
| amt + ants.r + ant.h + other.h + all.h + soil + s.temp*s.time | 406.13 | 6.30 | 3.44E-03 |
| ants.r * ant.h + other.h + all.h + sage + s.temp*s.time | 406.19 | 6.36 | 3.34E-03 |
| amt + ants.r * ant.h + sage + s.temp*s.time | 406.21 | 6.37 | 3.31E-03 |
| ants.r * ant.h + all.h + sage + soil + s.temp*s.time | 406.30 | 6.47 | 3.16E-03 |
| amt + amp + ants.r * ant.h + all.h + sage + s.temp*s.time | 406.35 | 6.51 | 3.09E-03 |
| amt*amp + ants.r + other.h + s.temp*s.time | 406.41 | 6.57 | 3.00E-03 |
| ants.r * ant.h + other.h + s.temp*s.time | 406.42 | 6.58 | 2.99E-03 |
| ants.r * ant.h + soil + s.temp*s.time | 406.46 | 6.62 | 2.92E-03 |
| ants.r + other.h + all.h + sage + soil + s.temp*s.time | 406.47 | 6.63 | 2.91E-03 |
| ants.r * ant.h + sage + s.temp*s.time | 406.50 | 6.66 | 2.86E-03 |
| ants.r * ant.h + other.h + all.h + soil + s.temp*s.time | 406.53 | 6.69 | 2.82E-03 |
| amt*amp + ants.r + soil + s.temp*s.time | 406.53 | 6.70 | 2.81E-03 |
| amt*amp + ants.r + sage + s.temp*s.time | 406.54 | 6.70 | 2.81E-03 |
| amt*amp + ants.r + ant.h + s.temp*s.time | 406.55 | 6.71 | 2.79E-03 |
| amt + ants.r + ant.h + all.h + sage + soil + s.temp*s.time | 406.65 | 6.81 | 2.66E-03 |
| amt + ants.r + ant.h + sage + s.temp*s.time | 406.65 | 6.82 | 2.65E-03 |
| amt*amp + ants.r + all.h + sage + s.temp*s.time | 406.78 | 6.94 | 2.49E-03 |
| amt*amp + ants.r + other.h + all.h + s.temp*s.time | 406.84 | 7.00 | 2.42E-03 |
| amt*amp + ants.r + all.h + soil + s.temp*s.time | 406.87 | 7.03 | 2.39E-03 |
| amt + amp + ants.r + ant.h + other.h + all.h + sage + s.temp*s.time | 406.92 | 7.09 | 2.32E-03 |
| ants.r + other.h + sage + soil + s.temp*s.time | 407.01 | 7.17 | 2.22E-03 |
| amt + amp + ants.r + all.h + s.temp*s.time | 407.08 | 7.25 | 2.14E-03 |
| amt + ants.r * ant.h + other.h + sage + soil + s.temp*s.time | 407.11 | 7.27 | 2.11E-03 |
| ants.r + ant.h + sage + soil + s.temp*s.time | 407.19 | 7.35 | 2.03E-03 |
| amt + ant.h + s.temp*s.time | 407.19 | 7.35 | 2.03E-03 |
| amt + amp + ants.r * ant.h + sage + s.temp*s.time | 407.20 | 7.36 | 2.02E-03 |
| amt + ants.r + ant.h + all.h + sage + s.temp*s.time | 407.23 | 7.40 | 1.98E-03 |
| ants.r + ant.h + other.h + sage + s.temp*s.time | 407.25 | 7.41 | 1.97E-03 |
| ants.r + ant.h + other.h + soil + s.temp*s.time | 407.48 | 7.65 | 1.75E-03 |
| amt + amp + ants.r + other.h + all.h + soil + s.temp*s.time | 407.62 | 7.79 | 1.63E-03 |
| amt + ants.r * ant.h + other.h + sage + s.temp*s.time | 407.63 | 7.79 | 1.63E-03 |
| amt + ants.r * ant.h + other.h + all.h + sage + s.temp*s.time | 407.64 | 7.80 | 1.62E-03 |
| amt + ants.r * ant.h + all.h + sage + soil + s.temp*s.time | 407.66 | 7.83 | 1.60E-03 |
| amt + amp + ants.r + other.h + sage + s.temp*s.time | 407.67 | 7.83 | 1.60E-03 |
| amt*amp + ants.r * ant.h + s.temp*s.time | 407.69 | 7.85 | 1.58E-03 |
| amt + amp + ants.r + ant.h + other.h + s.temp*s.time | 407.69 | 7.86 | 1.58E-03 |
| amt + amp + ants.r + ant.h + all.h + sage + soil + s.temp*s.time | 407.74 | 7.90 | 1.54E-03 |
| ants.r + ant.h + other.h + all.h + sage + soil + s.temp*s.time | 407.80 | 7.96 | 1.50E-03 |
| amt + sage + soil + s.temp*s.time | 407.82 | 7.98 | 1.48E-03 |
| amt + ants.r * ant.h + s.temp*s.time | 407.82 | 7.98 | 1.48E-03 |
| amt + amp + ant.h + soil + s.temp*s.time | 407.85 | 8.01 | 1.46E-03 |
| amt + ants.r + ant.h + other.h + all.h + s.temp*s.time | 407.88 | 8.05 | 1.43E-03 |
| amt*amp + ants.r + ant.h + other.h + all.h + s.temp*s.time | 407.95 | 8.11 | 1.39E-03 |
| amt + ants.r * ant.h + other.h + s.temp*s.time | 407.98 | 8.14 | 1.37E-03 |
| amt + ants.r + ant.h + other.h + all.h + sage + soil + s.temp*s.time | 408.00 | 8.16 | 1.35E-03 |
| amt + amp + ants.r + ant.h + other.h + sage + s.temp*s.time | 408.00 | 8.16 | 1.35E-03 |
| amt + amp + ants.r + ant.h + sage + soil + s.temp*s.time | 408.02 | 8.18 | 1.34E-03 |
| amt + amp + ants.r + other.h + all.h + s.temp*s.time | 408.02 | 8.18 | 1.34E-03 |
| amt + ants.r + ant.h + other.h + s.temp*s.time | 408.09 | 8.25 | 1.30E-03 |
| amt*amp + ants.r * ant.h + other.h + all.h + s.temp*s.time | 408.11 | 8.28 | 1.28E-03 |
| amt + amp + ants.r + ant.h + s.temp*s.time | 408.15 | 8.32 | 1.25E-03 |
| amt + amp + ants.r * ant.h + other.h + all.h + sage + soil + s.temp*s.time | 408.19 | 8.36 | 1.23E-03 |
| amt + amp + ants.r * ant.h + s.temp*s.time | 408.24 | 8.40 | 1.20E-03 |
| amt + other.h + s.temp*s.time | 408.35 | 8.52 | 1.13E-03 |
| amt*amp + ants.r + ant.h + all.h + sage + s.temp*s.time | 408.37 | 8.54 | 1.12E-03 |
| amt*amp + ants.r * ant.h + all.h + sage + s.temp*s.time | 408.46 | 8.62 | 1.08E-03 |
| amt*amp + ants.r + ant.h + all.h + soil + s.temp*s.time | 408.50 | 8.66 | 1.05E-03 |
| ants.r * ant.h + other.h + sage + s.temp*s.time | 408.52 | 8.68 | 1.04E-03 |
| ants.r * ant.h + sage + soil + s.temp*s.time | 408.57 | 8.74 | 1.02E-03 |
| amt*amp + ants.r * ant.h + all.h + soil + s.temp*s.time | 408.62 | 8.79 | 9.90E-04 |
| ants.r * ant.h + other.h + all.h + sage + soil + s.temp*s.time | 408.63 | 8.80 | 9.85E-04 |
| amt + ants.r + ant.h + other.h + all.h + sage + s.temp*s.time | 408.64 | 8.80 | 9.81E-04 |
| ants.r * ant.h + other.h + soil + s.temp*s.time | 408.80 | 8.97 | 9.06E-04 |
| amt*amp + ants.r + other.h + sage + s.temp*s.time | 408.97 | 9.13 | 8.33E-04 |
| amt + amp + ants.r * ant.h + all.h + sage + soil + s.temp*s.time | 408.99 | 9.16 | 8.24E-04 |
| amt*amp + ants.r + ant.h + other.h + s.temp*s.time | 409.00 | 9.16 | 8.20E-04 |
| amt*amp + ants.r + other.h + soil + s.temp*s.time | 409.01 | 9.17 | 8.18E-04 |
| amt*amp + ants.r + sage + soil + s.temp*s.time | 409.10 | 9.26 | 7.81E-04 |
| amt*amp + ants.r + ant.h + soil + s.temp*s.time | 409.14 | 9.30 | 7.66E-04 |
| amt*amp + ants.r + ant.h + sage + s.temp*s.time | 409.14 | 9.30 | 7.66E-04 |
| amt + amp + ants.r + ant.h + all.h + s.temp*s.time | 409.17 | 9.33 | 7.54E-04 |
| amt + ants.r + all.h + s.temp*s.time | 409.20 | 9.36 | 7.43E-04 |
| amt*amp + ants.r + all.h + sage + soil + s.temp*s.time | 409.42 | 9.59 | 6.64E-04 |
| amt*amp + ants.r + other.h + all.h + sage + s.temp*s.time | 409.43 | 9.59 | 6.62E-04 |
| amt + ants.r * ant.h + other.h + all.h + s.temp*s.time | 409.44 | 9.60 | 6.60E-04 |
| amt + amp + ants.r * ant.h + other.h + s.temp*s.time | 409.45 | 9.61 | 6.57E-04 |
| ants.r + ant.h + other.h + sage + soil + s.temp*s.time | 409.45 | 9.61 | 6.55E-04 |
| amt + ants.r * ant.h + all.h + s.temp*s.time | 409.50 | 9.66 | 6.40E-04 |
| amt*amp + ants.r + other.h + all.h + soil + s.temp*s.time | 409.51 | 9.68 | 6.35E-04 |
| amt + amp + ants.r + ant.h + other.h + all.h + sage + soil + s.temp*s.time | 409.58 | 9.75 | 6.13E-04 |
| amt + amp + ants.r + all.h + soil + s.temp*s.time | 409.73 | 9.89 | 5.70E-04 |
| amt + amp + ants.r * ant.h + other.h + sage + s.temp*s.time | 409.73 | 9.90 | 5.68E-04 |
| amt + amp + ants.r * ant.h + sage + soil + s.temp*s.time | 409.80 | 9.96 | 5.50E-04 |
| amt*amp + ants.r * ant.h + other.h + s.temp*s.time | 409.97 | 10.13 | 5.05E-04 |
| amt + amp + ants.r + ant.h + other.h + all.h + s.temp*s.time | 410.19 | 10.35 | 4.53E-04 |
| amt + amp + ants.r + other.h + all.h + sage + s.temp*s.time | 410.19 | 10.36 | 4.52E-04 |
| amt + ants.r * ant.h + other.h + all.h + sage + soil + s.temp*s.time | 410.20 | 10.36 | 4.51E-04 |
| amt + amp + ants.r + ant.h + other.h + soil + s.temp*s.time | 410.25 | 10.41 | 4.40E-04 |
| amt + ants.r + ant.h + s.temp*s.time | 410.25 | 10.42 | 4.38E-04 |
| amt*amp + ants.r * ant.h + soil + s.temp*s.time | 410.27 | 10.43 | 4.35E-04 |
| amt*amp + ants.r * ant.h + sage + s.temp*s.time | 410.35 | 10.51 | 4.18E-04 |
| amt + amp + ants.r * ant.h + all.h + s.temp*s.time | 410.37 | 10.53 | 4.14E-04 |
| amt + ants.r + ant.h + all.h + s.temp*s.time | 410.41 | 10.57 | 4.05E-04 |
| amt + amp + ants.r * ant.h + soil + s.temp*s.time | 410.47 | 10.63 | 3.94E-04 |
| amt + amp + ants.r + ant.h + other.h + sage + soil + s.temp*s.time | 410.59 | 10.76 | 3.70E-04 |
| amt*amp + ants.r + ant.h + other.h + all.h + sage + s.temp*s.time | 410.64 | 10.80 | 3.61E-04 |
| amt*amp + ants.r + ant.h + other.h + all.h + soil + s.temp*s.time | 410.68 | 10.84 | 3.54E-04 |
| ants.r * ant.h + other.h + sage + soil + s.temp*s.time | 410.71 | 10.87 | 3.49E-04 |
| amt + amp + ants.r + ant.h + soil + s.temp*s.time | 410.80 | 10.96 | 3.33E-04 |
| amt*amp + ants.r * ant.h + other.h + all.h + soil + s.temp*s.time | 410.86 | 11.02 | 3.24E-04 |
| amt*amp + ants.r * ant.h + other.h + all.h + sage + s.temp*s.time | 410.88 | 11.04 | 3.21E-04 |
| amt + amp + sage + s.temp*s.time | 410.93 | 11.09 | 3.12E-04 |
| amt*amp + ants.r + ant.h + all.h + sage + soil + s.temp*s.time | 411.10 | 11.26 | 2.88E-04 |
| amt*amp + ants.r * ant.h + all.h + sage + soil + s.temp*s.time | 411.15 | 11.31 | 2.80E-04 |
| amt*amp + ants.r + other.h + sage + soil + s.temp*s.time | 411.60 | 11.76 | 2.23E-04 |
| amt*amp + ants.r + ant.h + other.h + sage + s.temp*s.time | 411.64 | 11.80 | 2.20E-04 |
| all.h + s.temp*s.time | 411.65 | 11.81 | 2.19E-04 |
| all.h + s.temp*s.time | 411.65 | 11.81 | 2.19E-04 |
| amt*amp + ants.r + ant.h + other.h + soil + s.temp*s.time | 411.67 | 11.84 | 2.15E-04 |
| amt*amp + ants.r + ant.h + sage + soil + s.temp*s.time | 411.77 | 11.93 | 2.06E-04 |
| amt + amp + ants.r * ant.h + other.h + all.h + s.temp*s.time | 411.90 | 12.06 | 1.92E-04 |
| amt + ants.r * ant.h + all.h + soil + s.temp*s.time | 411.91 | 12.07 | 1.92E-04 |
| amt + amp + ants.r + ant.h + all.h + soil + s.temp*s.time | 411.91 | 12.07 | 1.92E-04 |
| amt + amp + ants.r * ant.h + other.h + soil + s.temp*s.time | 412.09 | 12.25 | 1.75E-04 |
| amt*amp + ants.r + other.h + all.h + sage + soil + s.temp*s.time | 412.15 | 12.31 | 1.70E-04 |
| amt + amp + ants.r * ant.h + other.h + sage + soil + s.temp*s.time | 412.40 | 12.56 | 1.50E-04 |
| amt*amp + ants.r * ant.h + other.h + sage + s.temp*s.time | 412.67 | 12.83 | 1.31E-04 |
| amt*amp + ants.r * ant.h + other.h + soil + s.temp*s.time | 412.68 | 12.84 | 1.31E-04 |
| amt + amp + ants.r + ant.h + other.h + all.h + soil + s.temp*s.time | 412.82 | 12.98 | 1.21E-04 |
| amt*amp + ants.r * ant.h + sage + soil + s.temp*s.time | 412.97 | 13.13 | 1.13E-04 |
| amt + amp + ants.r * ant.h + all.h + soil + s.temp*s.time | 413.06 | 13.23 | 1.08E-04 |
| amt*amp + ants.r + ant.h + other.h + all.h + sage + soil + s.temp*s.time | 413.43 | 13.59 | 8.96E-05 |
| amt*amp + ants.r * ant.h + other.h + all.h + sage + soil + s.temp*s.time | 413.61 | 13.78 | 8.17E-05 |
| ant.h + all.h + s.temp*s.time | 413.62 | 13.78 | 8.16E-05 |
| all.h + sage + s.temp*s.time | 413.69 | 13.85 | 7.88E-05 |
| all.h + soil + s.temp*s.time | 413.78 | 13.94 | 7.53E-05 |
| other.h + all.h + s.temp*s.time | 413.98 | 14.14 | 6.82E-05 |
| amt*amp + ants.r + ant.h + other.h + sage + soil + s.temp*s.time | 413.98 | 14.14 | 6.80E-05 |
| amt + amp + ants.r * ant.h + other.h + all.h + sage + s.temp*s.time | 414.33 | 14.49 | 5.71E-05 |
| amt*amp + all.h + s.temp*s.time | 414.55 | 14.71 | 5.13E-05 |
| other.h + s.temp*s.time | 414.77 | 14.93 | 4.58E-05 |
| ant.h + s.temp*s.time | 415.10 | 15.26 | 3.88E-05 |
| amt*amp + ants.r * ant.h + other.h + sage + soil + s.temp*s.time | 415.19 | 15.35 | 3.72E-05 |
| soil + s.temp*s.time | 415.38 | 15.54 | 3.38E-05 |
| sage + s.temp*s.time | 415.44 | 15.60 | 3.29E-05 |
| amt + amp + all.h + sage + s.temp*s.time | 415.45 | 15.61 | 3.27E-05 |
| ant.h + all.h + sage + s.temp*s.time | 415.65 | 15.81 | 2.95E-05 |
| ant.h + all.h + soil + s.temp*s.time | 415.67 | 15.83 | 2.93E-05 |
| ant.h + other.h + all.h + s.temp*s.time | 415.75 | 15.91 | 2.81E-05 |
| amt*amp + s.temp*s.time | 415.83 | 15.99 | 2.70E-05 |
| amt + all.h + soil + s.temp*s.time | 415.96 | 16.12 | 2.53E-05 |
| all.h + sage + soil + s.temp*s.time | 415.99 | 16.16 | 2.49E-05 |
| amt + amp + s.temp*s.time | 416.01 | 16.17 | 2.47E-05 |
| other.h + all.h + sage + s.temp*s.time | 416.10 | 16.26 | 2.36E-05 |
| other.h + all.h + soil + s.temp*s.time | 416.10 | 16.26 | 2.36E-05 |
| amt + amp + ants.r * ant.h + other.h + all.h + soil + s.temp*s.time | 416.18 | 16.35 | 2.26E-05 |
| amt*amp + ant.h + all.h + s.temp*s.time | 416.19 | 16.35 | 2.26E-05 |
| amt + ants.r + soil + s.temp*s.time | 416.33 | 16.49 | 2.10E-05 |
| amt + amp + sage + soil + s.temp*s.time | 416.38 | 16.54 | 2.05E-05 |
| amt + other.h + soil + s.temp*s.time | 416.88 | 17.04 | 1.59E-05 |
| ant.h + other.h + s.temp*s.time | 416.88 | 17.04 | 1.59E-05 |
| other.h + sage + s.temp*s.time | 417.05 | 17.21 | 1.47E-05 |
| amt*amp + other.h + all.h + s.temp*s.time | 417.23 | 17.39 | 1.34E-05 |
| amt*amp + all.h + soil + s.temp*s.time | 417.28 | 17.45 | 1.30E-05 |
| amt*amp + all.h + sage + s.temp*s.time | 417.29 | 17.46 | 1.30E-05 |
| other.h + soil + s.temp*s.time | 417.30 | 17.47 | 1.29E-05 |
| amt + other.h + all.h + soil + s.temp*s.time | 417.33 | 17.49 | 1.28E-05 |
| ant.h + sage + s.temp*s.time | 417.43 | 17.59 | 1.21E-05 |
| ant.h + soil + s.temp*s.time | 417.47 | 17.64 | 1.19E-05 |
| amt + ant.h + other.h + soil + s.temp*s.time | 417.47 | 17.64 | 1.19E-05 |
| amt + amp + ant.h + all.h + sage + s.temp*s.time | 417.48 | 17.64 | 1.18E-05 |
| amt + other.h + sage + s.temp*s.time | 417.48 | 17.64 | 1.18E-05 |
| amt + other.h + sage + soil + s.temp*s.time | 417.59 | 17.75 | 1.12E-05 |
| amt*amp + other.h + s.temp*s.time | 417.67 | 17.83 | 1.07E-05 |
| sage + soil + s.temp*s.time | 417.69 | 17.85 | 1.07E-05 |
| ant.h + all.h + sage + soil + s.temp*s.time | 417.74 | 17.90 | 1.04E-05 |
| ant.h + other.h + all.h + sage + s.temp*s.time | 417.77 | 17.93 | 1.02E-05 |
| ant.h + other.h + all.h + soil + s.temp*s.time | 418.02 | 18.18 | 9.02E-06 |
| amt + amp + other.h + all.h + sage + soil + s.temp*s.time | 418.06 | 18.23 | 8.83E-06 |
| amt + amp + all.h + sage + soil + s.temp*s.time | 418.07 | 18.23 | 8.81E-06 |
| amt + all.h + sage + soil + s.temp*s.time | 418.08 | 18.24 | 8.77E-06 |
| amt + all.h + sage + s.temp*s.time | 418.09 | 18.25 | 8.72E-06 |
| amt + ant.h + all.h + soil + s.temp*s.time | 418.13 | 18.30 | 8.52E-06 |
| amt*amp + soil + s.temp*s.time | 418.22 | 18.38 | 8.18E-06 |
| amt*amp + ant.h + s.temp*s.time | 418.29 | 18.45 | 7.90E-06 |
| amt*amp + sage + s.temp*s.time | 418.35 | 18.51 | 7.65E-06 |
| other.h + all.h + sage + soil + s.temp*s.time | 418.38 | 18.55 | 7.53E-06 |
| amt + amp + soil + s.temp*s.time | 418.41 | 18.58 | 7.41E-06 |
| amt + ants.r + sage + soil + s.temp*s.time | 418.48 | 18.64 | 7.17E-06 |
| amt + ants.r + sage + s.temp*s.time | 418.50 | 18.66 | 7.10E-06 |
| amt*amp + ant.h + other.h + all.h + s.temp*s.time | 418.57 | 18.73 | 6.87E-06 |
| amt*amp + ant.h + all.h + sage + s.temp*s.time | 418.66 | 18.82 | 6.57E-06 |
| amt*amp + ant.h + all.h + soil + s.temp*s.time | 418.75 | 18.92 | 6.26E-06 |
| amt + amp + other.h + soil + s.temp*s.time | 418.86 | 19.02 | 5.94E-06 |
| amt + amp + other.h + sage + soil + s.temp*s.time | 418.88 | 19.04 | 5.87E-06 |
| amt + amp + ant.h + sage + s.temp*s.time | 418.97 | 19.13 | 5.61E-06 |
| ant.h + other.h + sage + s.temp*s.time | 419.07 | 19.23 | 5.35E-06 |
| amt + amp + other.h + s.temp*s.time | 419.11 | 19.27 | 5.24E-06 |
| ant.h + other.h + soil + s.temp*s.time | 419.18 | 19.34 | 5.06E-06 |
| amt + amp + all.h + s.temp*s.time | 419.29 | 19.46 | 4.77E-06 |
| amt + ant.h + other.h + all.h + soil + s.temp*s.time | 419.33 | 19.49 | 4.69E-06 |
| amt + other.h + all.h + sage + soil + s.temp*s.time | 419.36 | 19.52 | 4.62E-06 |
| other.h + sage + soil + s.temp*s.time | 419.45 | 19.61 | 4.41E-06 |
| amt + other.h + all.h + sage + s.temp*s.time | 419.59 | 19.75 | 4.12E-06 |
| amt + amp + ant.h + other.h + all.h + sage + s.temp*s.time | 419.60 | 19.76 | 4.11E-06 |
| amt + ant.h + other.h + sage + soil + s.temp*s.time | 419.69 | 19.85 | 3.92E-06 |
| amt + ant.h + other.h + sage + s.temp*s.time | 419.75 | 19.91 | 3.80E-06 |
| ant.h + sage + soil + s.temp*s.time | 419.80 | 19.96 | 3.71E-06 |
| amt*amp + all.h + sage + soil + s.temp*s.time | 419.80 | 19.96 | 3.70E-06 |
| amt*amp + other.h + all.h + soil + s.temp*s.time | 419.86 | 20.02 | 3.60E-06 |
| amt*amp + other.h + all.h + sage + s.temp*s.time | 419.86 | 20.03 | 3.59E-06 |
| amt + amp + ants.r + soil + s.temp*s.time | 419.87 | 20.04 | 3.57E-06 |
| amt + other.h + all.h + s.temp*s.time | 419.93 | 20.09 | 3.48E-06 |
| amt + amp + ant.h + all.h + sage + soil + s.temp*s.time | 419.97 | 20.14 | 3.40E-06 |
| amt*amp + ant.h + other.h + s.temp*s.time | 420.01 | 20.17 | 3.34E-06 |
| amt*amp + other.h + soil + s.temp*s.time | 420.04 | 20.21 | 3.28E-06 |
| amt*amp + other.h + sage + s.temp*s.time | 420.08 | 20.25 | 3.22E-06 |
| ant.h + other.h + all.h + sage + soil + s.temp*s.time | 420.15 | 20.32 | 3.11E-06 |
| amt + ant.h + all.h + sage + soil + s.temp*s.time | 420.25 | 20.41 | 2.97E-06 |
| amt + amp + ants.r + s.temp*s.time | 420.27 | 20.43 | 2.93E-06 |
| amt + ant.h + all.h + sage + s.temp*s.time | 420.48 | 20.64 | 2.64E-06 |
| amt*amp + ant.h + soil + s.temp*s.time | 420.56 | 20.72 | 2.54E-06 |
| amt*amp + sage + soil + s.temp*s.time | 420.59 | 20.76 | 2.49E-06 |
| amt*amp + ant.h + sage + s.temp*s.time | 420.60 | 20.76 | 2.49E-06 |
| amt + amp + other.h + all.h + soil + s.temp*s.time | 420.60 | 20.76 | 2.48E-06 |
| amt + ants.r + other.h + s.temp*s.time | 420.84 | 21.00 | 2.20E-06 |
| amt + amp + ant.h + other.h + s.temp*s.time | 420.89 | 21.05 | 2.15E-06 |
| amt + amp + ant.h + other.h + sage + s.temp*s.time | 420.90 | 21.06 | 2.14E-06 |
| amt + amp + other.h + sage + s.temp*s.time | 420.99 | 21.15 | 2.05E-06 |
| amt*amp + ant.h + other.h + all.h + soil + s.temp*s.time | 421.02 | 21.18 | 2.02E-06 |
| amt*amp + ant.h + other.h + all.h + sage + s.temp*s.time | 421.26 | 21.42 | 1.79E-06 |
| amt*amp + ant.h + all.h + sage + soil + s.temp*s.time | 421.27 | 21.44 | 1.77E-06 |
| amt + amp + other.h + all.h + s.temp*s.time | 421.37 | 21.53 | 1.69E-06 |
| amt + amp + ant.h + sage + soil + s.temp*s.time | 421.40 | 21.57 | 1.66E-06 |
| ant.h + other.h + sage + soil + s.temp*s.time | 421.42 | 21.58 | 1.65E-06 |
| amt + ant.h + other.h + all.h + sage + soil + s.temp*s.time | 421.49 | 21.65 | 1.59E-06 |
| amt + ant.h + other.h + all.h + sage + s.temp*s.time | 421.53 | 21.69 | 1.56E-06 |
| amt + amp + ant.h + all.h + s.temp*s.time | 421.58 | 21.74 | 1.52E-06 |
| amt + amp + all.h + soil + s.temp*s.time | 421.61 | 21.77 | 1.50E-06 |
| amt + ants.r + s.temp*s.time | 421.62 | 21.78 | 1.49E-06 |
| amt + ant.h + other.h + all.h + s.temp*s.time | 421.77 | 21.94 | 1.38E-06 |
| amt + amp + ant.h + other.h + all.h + sage + soil + s.temp*s.time | 421.78 | 21.94 | 1.38E-06 |
| amt + amp + ants.r + sage + s.temp*s.time | 421.92 | 22.08 | 1.28E-06 |
| amt*amp + other.h + all.h + sage + soil + s.temp*s.time | 422.00 | 22.16 | 1.24E-06 |
| amt*amp + ant.h + other.h + soil + s.temp*s.time | 422.22 | 22.39 | 1.10E-06 |
| amt*amp + ant.h + other.h + sage + s.temp*s.time | 422.34 | 22.50 | 1.04E-06 |
| amt*amp + other.h + sage + soil + s.temp*s.time | 422.57 | 22.73 | 9.28E-07 |
| amt + all.h + s.temp*s.time | 422.60 | 22.76 | 9.16E-07 |
| amt + amp + ant.h + other.h + all.h + s.temp*s.time | 422.73 | 22.90 | 8.54E-07 |
| amt*amp + ant.h + sage + soil + s.temp*s.time | 422.75 | 22.91 | 8.50E-07 |
| amt + amp + other.h + all.h + sage + s.temp*s.time | 422.83 | 23.00 | 8.13E-07 |
| amt + amp + ant.h + other.h + soil + s.temp*s.time | 423.08 | 23.24 | 7.20E-07 |
| amt + amp + ant.h + other.h + sage + soil + s.temp*s.time | 423.22 | 23.39 | 6.69E-07 |
| amt + ant.h + all.h + s.temp*s.time | 423.46 | 23.62 | 5.94E-07 |
| amt*amp + ant.h + other.h + all.h + sage + soil + s.temp*s.time | 423.46 | 23.63 | 5.93E-07 |
| amt + amp + ant.h + all.h + soil + s.temp*s.time | 423.72 | 23.88 | 5.22E-07 |
| amt*amp + ant.h + other.h + sage + soil + s.temp*s.time | 423.79 | 23.96 | 5.03E-07 |
| amt + amp + ant.h + other.h + all.h + soil + s.temp*s.time | 424.10 | 24.26 | 4.32E-07 |
| amt + sage + s.temp*s.time | 424.10 | 24.27 | 4.31E-07 |
| amt + soil + s.temp*s.time | 424.41 | 24.57 | 3.70E-07 |
| amt + amp + s.temp*s.time | 425.37 | 25.53 | 2.29E-07 |
| amt + ants.r * ant.h + other.h + all.h + soil + s.temp*s.time | 425.80 | 25.97 | 1.84E-07 |

***^a^*** The environmental variables included in these models are annual mean temperature (amt), annual mean precipitation (amp), ant mound density per hectare (ants), diversity of non-ant arthropods in pitfall traps (other.h), diversity of ants in pitfall traps (ant.h), percentage of ground area that was sage (sage), survey start time (s.time), and temperature at the start of each survey (s.temp)

**Table S6.** Predictions for top three models. Relationships between top environmental predictors and response variable (probability of plot occupancy or estimated abundance of lizards). The best model predicting probability of plot occupancy included annual mean temperature (amt), annual mean precipitation (amp), density of ant mounds per hectare (ants), and the Shannon diversity of available ants in the plot (antH). The best model to estimate lizard abundance included temperature, precipitation, and density of ant mounds. To calculate the estimates for each predictor variable, we made predictions of the response variable across the observed range of values for that variable (“focal predictor”) and held other conditions (“interacting predictors”) constant at mean values “(m),” high values (“(h)” = 3^rd^ quartile of variable range), and low values (“(l)” = 1^st^ quartile of variable range). We report the range of the response over the range of the focal predictor variable for the model conditions that gave the greatest response. The sign of the relationship (“Sign”) is the direction of the effect of each predictor variable on the response over the observed range of that variable.

| **Model rank** | **Predictor variable^a^** | **Response** | **Range of response** | **Range focal predictor** | **Value interacting predictor** | **r (abund)** | **Sign** |
| --- | --- | --- | --- | --- | --- | --- | --- |
| 1 | amt | Occupancy | 0.37 – 0.99 | 6.2 – 11.3 C | amp (h): 324 mm | na | + |
| 1 | amp | Occupancy | 0.99 – 0.37 | 193 – 345 mm | amt (h): 9.4 | na | - |
| 1 | ants | Occupancy | 0.49 – 1 | 0 – 25 mounds/ha | antH (l): 4E-6 | na | + |
| 1 | antH | Occupancy | 1 – 4E-3 | 0 – 0.67 H' | ants (h): 6.4 mounds/ha | na | - |
| 1 | amt | Abundance | 3.8–7.4 | 6.5 – 11.3 C | NA | -0.37 | - |
| 1 | amp | Abundance | 2.7–9.6 | 193 – 345 mm | NA | 0.49 | + |
| 1 | ants | Abundance | 4.7–11.1 | 0 – 25 mnds/ha | NA | 0.33 | + |
| 2 | amt | Occupancy | 0.97 – 1 | 6.2 – 11.3 C | amp (h): 234 mm | na | + |
| 2 | amp | Occupancy | 0.98 – 0.99 | 193 – 345 mm | amt (l): 7.2 | na | - |
| 2 | ants | Occupancy | 0.97 – 0.99 | 0 – 25 mounds/ha | antH (h): 0.46 | na | + |
| 2 | antH | Occupancy | 0.5 – 1 | 0 – 0.67 H' | ants (h): 6.4 mounds/ha | na | - |
| 2 | otherH | Occupancy | 0.99 – 0.99 | 0 – 0.82 H' | NA | na | - |
| 2 | amt | Abundance | 3.6–8.9 | 6.5 – 11.3 C | amp (h) 319 mm | -0.56 | - |
| 2 | amp | Abundance | 0.1–12.1 | 193 – 345 mm | amt (l) 9.4 | 0.14 | + |
| 2 | ants | Abundance | 3.6–8.9 | 0 – 25 mnds/ha | antH (l) 0 | 0.42 | + |
| 3 | amt | Occupancy | 0.33 – 0.98 | 6.2 – 11.3 C | amp (h): 324 mm | na | + |
| 3 | amp | Occupancy | 0.25 – 0.99 | 193 – 345 mm | amt (h): 9.4 | na | - |
| 3 | ants | Occupancy | 0.34 – 1 | 0 – 25 mounds/ha | antH (l): 4E-6 | na | + |
| 3 | amt | Abundance | 3.7–6.6 | 6.5 – 11.3 C | amp (m) 319 mm | -0.28 | - |
| 3 | amp | Abundance | 2.1–9.2 | 193 – 345 mm | amt (l) 7.3 | 0.49 | + |
| 3 | ants | Abundance | 4.2–10.5 | 0 – 25 mnds/ha | NA | 0.36 | + |
| 3 | sage | Abundance | 5–7 | 0 –43% | NA | -0.12 | - |

***^a^*** The environmental variables included in these models are annual mean temperature (amt), annual mean precipitation (amp), ant mound density per hectare (ants), diversity of non-ant arthropods in pitfall traps (other.h), diversity of ants in pitfall traps (ant.h), percentage of ground area that was sage (sage), survey start time (s.time), and temperature at the start of each survey (s.temp).

**
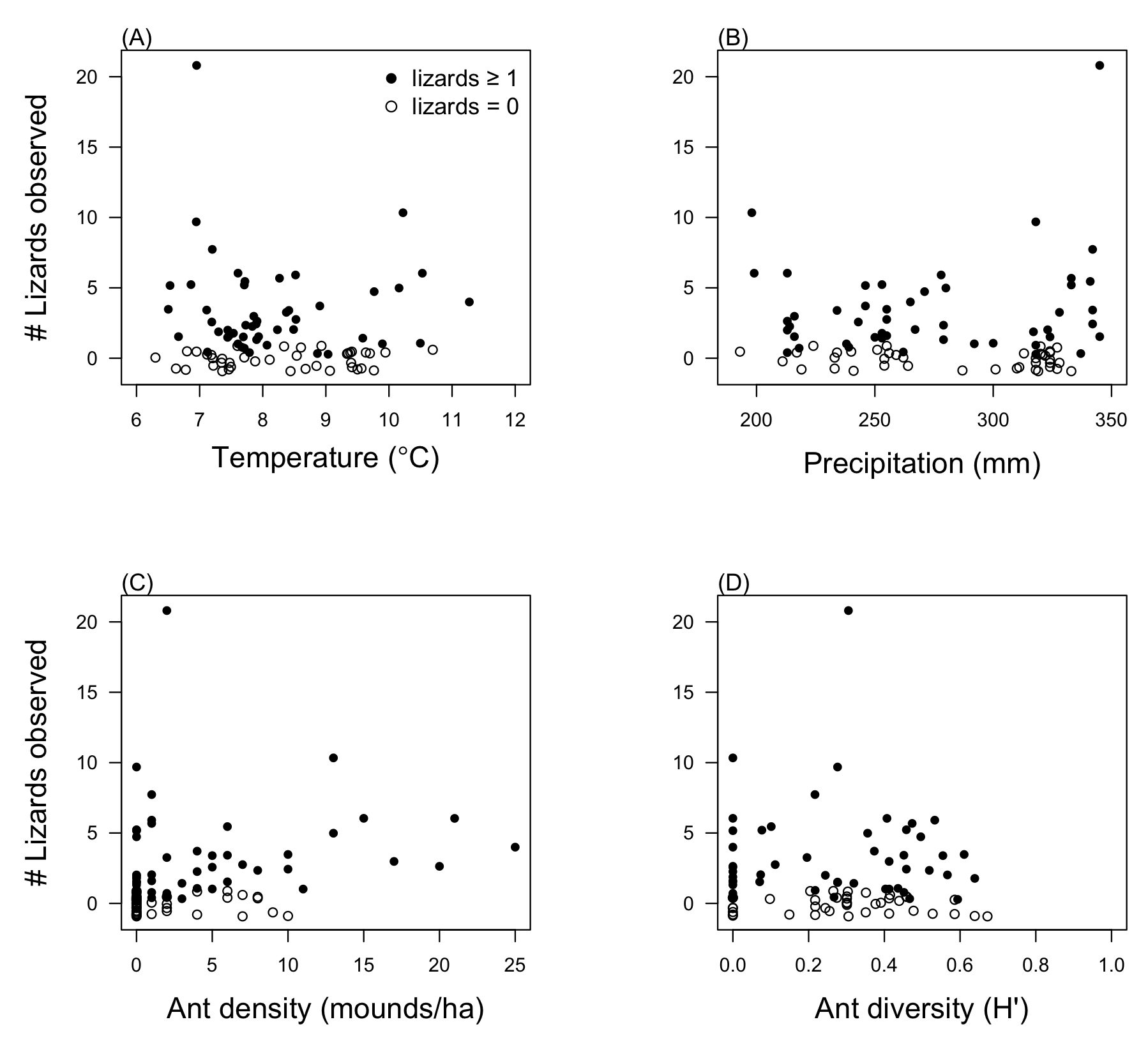
**

**Figure S2**. Raw lizard count data from 89 half-hectare plots in Wyoming, USA, was collected between June and September 2012 and 2013. Lizard numbers (including zeros; indicated by open circles) are offset to show overlapping points and are plotted against A) annual mean temperature, B) annual mean precipitation, C) ant mound density (mounds/ha), and D) ant diversity (H'). Only ant mound density was significantly related to raw lizard numbers (p = 0.04).


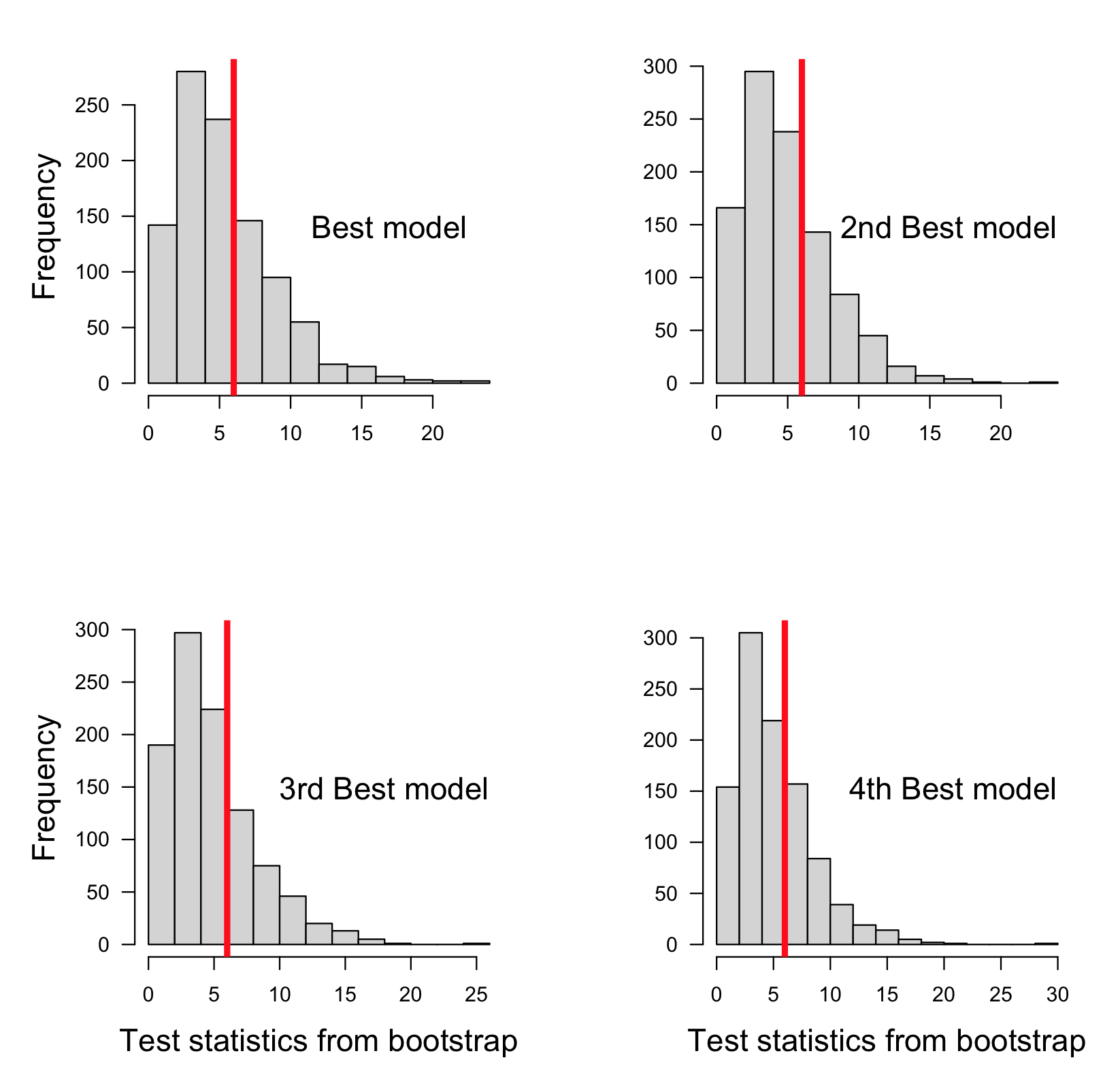


**Figure S3**. The goodness-of-fit of the best occupancy models were assessed using a chi-square test of observed and bootstrapped data, following the approach of MacKenzie and Bailey 2004. Models were considered a good fit if the observed test statistic (red line) was within the distribution of the bootstrapped test statistics; the observed test statistics were well within the distribution of bootstrap test results (P > 0.5) for the four best model.


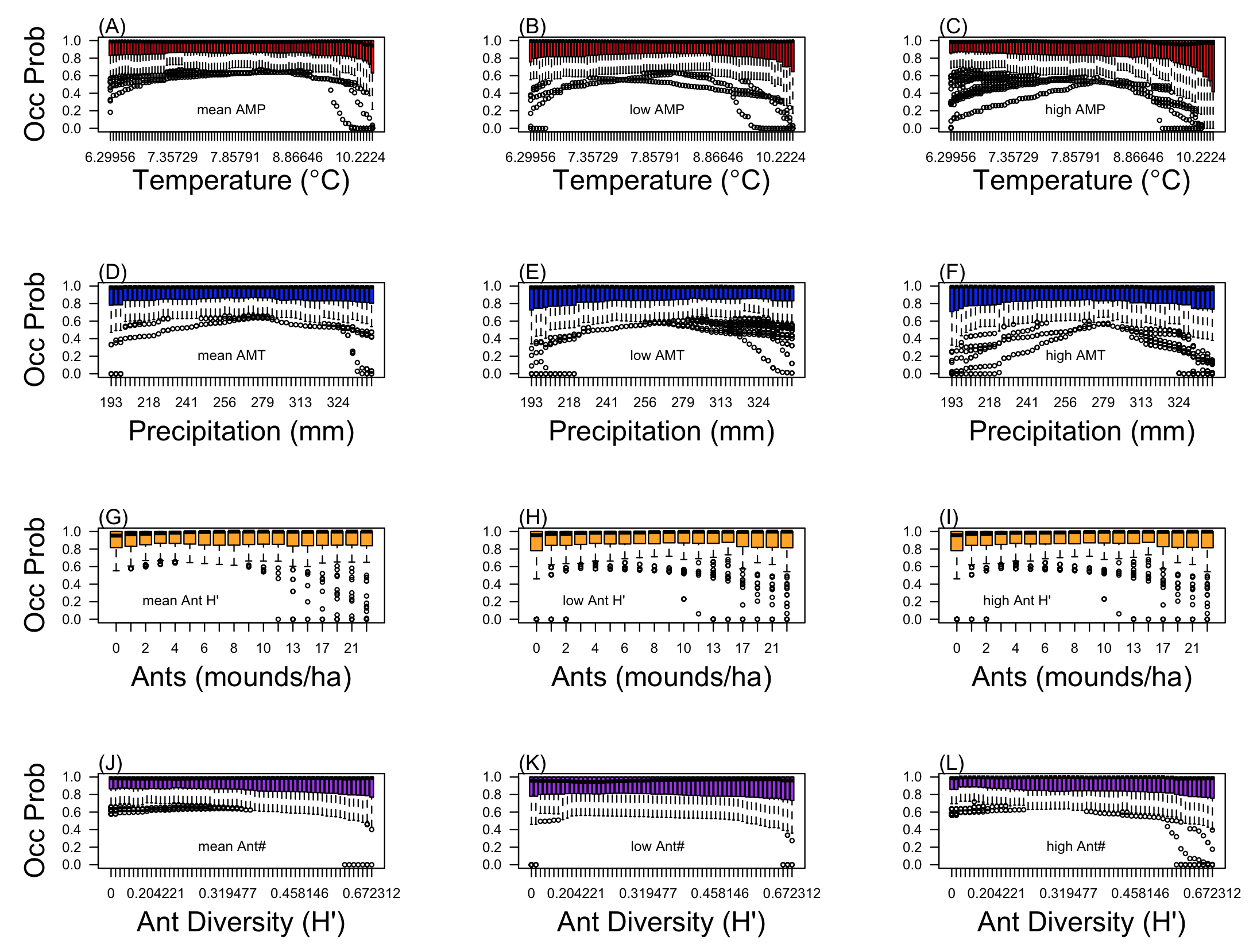


**Figure S4.** We used a bootstrap approach to generate 95% confidence intervals on occupancy predictions. We generated these estimates for each predictor variable—A–C) annual mean temperature, D–F) annual mean precipitation, G–I) Ant mound density, J–L) Ant diversity—across the observed range of values for that variable and held other conditions constant at mean values “(m),” high values (“(h)” = 3^rd^ quartile of variable range), and low values (“(l)” = 1^st^ quartile of variable range). Bootstrapping from a binomial distribution can produce results that are difficult to interpret, but the bootstrap of the data did not show a bias in our results. The results are, however, sensitive to getting a good sample across the distribution. Most sites we sampled had ant mounds, because that was part of our search criteria for potential lizard habitat. The bootstrap reflects that probability of lizard detection went up with ant mound > 0. Our results were sensitive to low numbers of ant mounds because we did not have many observations of low ant mound density. We therefore lacked power to detect effect of ant mounds on lizard density beyond ant mound >0.


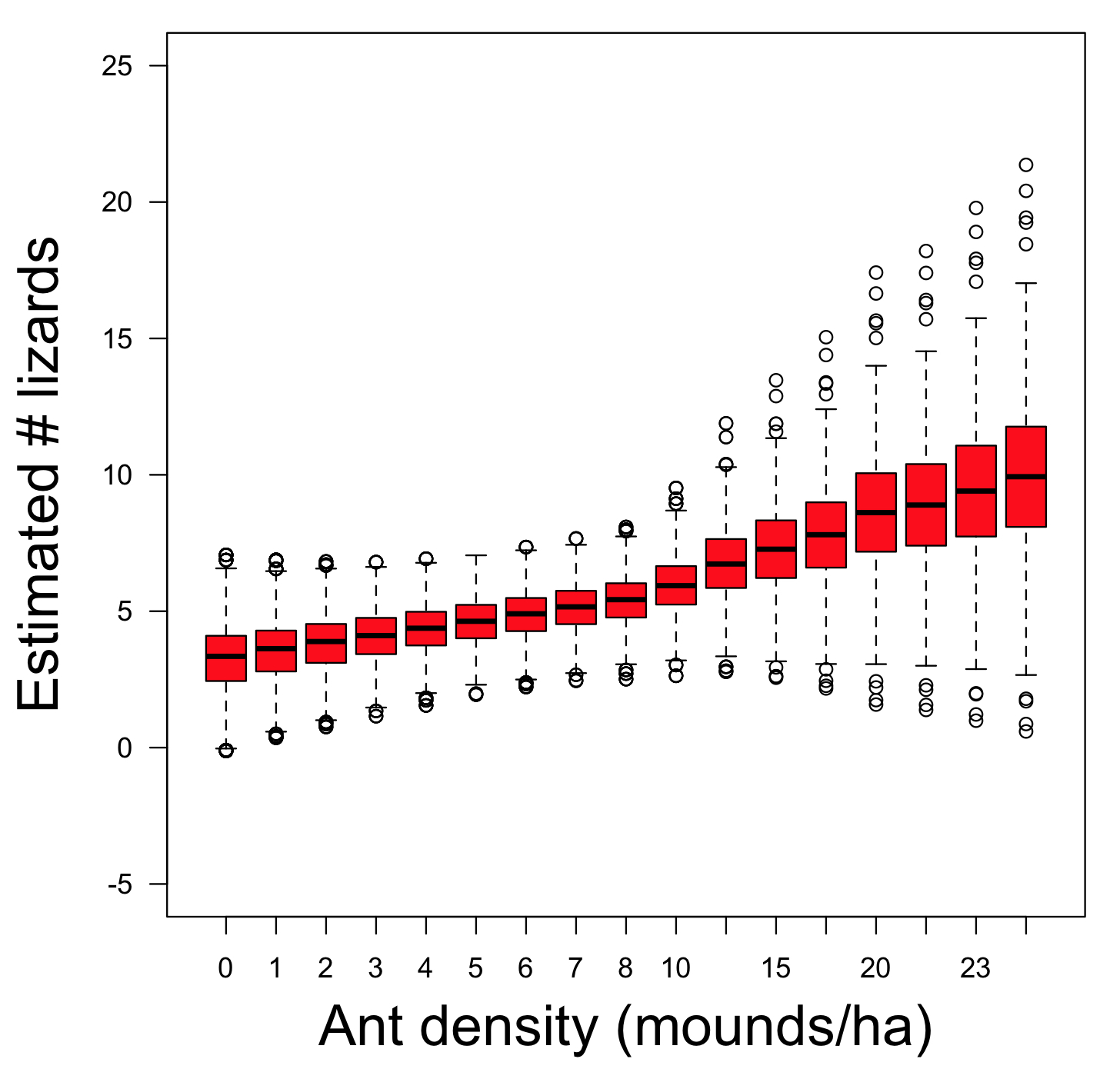


**Figure S5.** 95% confidence intervals on predicting lizard abundance using ant mound density.


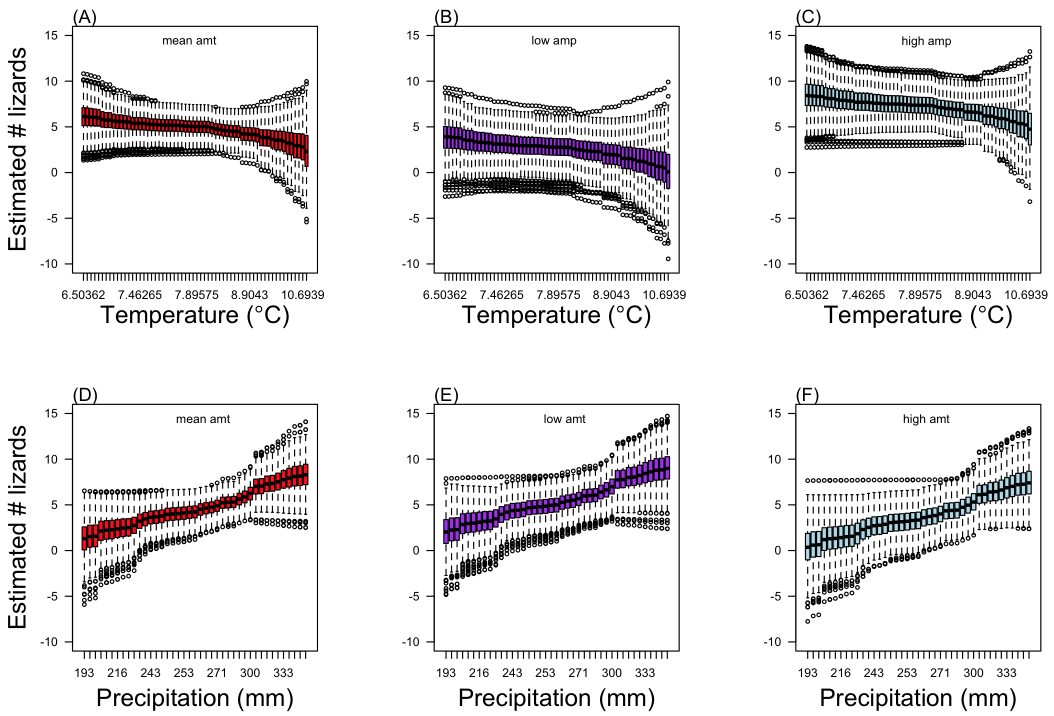


**Figure S6.** 95% confidence intervals on predicting lizard abundance using A–C) annual mean precipitation and D–F) annual mean temperature. We generated these estimates for each predictor variable across the observed range of values for that variable and held other conditions constant at mean values “(m),” high values (“(h)” = 3^rd^ quartile of variable range), and low values (“(l)” = 1^st^ quartile of variable range).
